# A single-cell atlas of ulcerative colitis reveals neutrophil–stromal circuits linked to biologic therapy resistance

**DOI:** 10.3389/fimmu.2026.1705328

**Published:** 2026-06-11

**Authors:** Shadi Toghi Eshghi, John Mark Gubatan, Parisa Mazrooei, Luis Quintanilla, Allen Nguyen, Amelia Au-Yeung, Derek R. Holman, Chikara Takahashi, Courtney Schiffman, William E. O’Gorman, Mary E. Keir, Saroja Ramanujan, Stephan Rogalla, Jason A. Hackney, Jacqueline M. McBride

**Affiliations:** 1 Computational Sciences, Research and Early Development, Genentech Inc., South San Francisco, CA, United States; 2 Division of Gastroenterology and Hepatology, Department of Medicine, Stanford University, Stanford, CA, United States; 3 Translational Medicine, Research and Early Development, Genentech Inc, South San Francisco, CA, United States; 4 Product Development, Data Sciences, Genentech Inc., South San Francisco, CA, United States; 5 Immunology Discovery, Research and Early Development, Genentech Inc, South San Francisco, CA, United States; 6 Preclinical & Translational Pharmacokinetics and Pharmacodynamics (PKPD), Genentech Inc., South San Francisco, CA, United States

**Keywords:** cell-cell communication, inflammatory fibroblasts, neutrophil heterogeneity, single cell RNA-sequencing (scRNA-seq), therapy resistance, transcriptomic profiling, ulcerative colitis

## Abstract

Ulcerative colitis (UC) is a chronic inflammatory bowel disease marked by immune cell infiltration, crypt erosion, and severe ulceration. In phase 3 studies with etrolizumab, the results of a transcriptional analysis of colonic biopsies revealed that etrolizumab-mediated integrin β7 blockade, but not adalimumab (a TNF-blocking antibody), reduced genes associated with integrin αEβ7+ intraepithelial lymphocytes (IELs). Both treatments significantly reduced stromal and myeloid cell-related genes linked to Mayo Clinic Score (MCS) remission status. A single-cell atlas from UC biopsies identified 36 distinct cell populations, including myeloid cells. This atlas enabled cell-specific signatures and cellular deconvolution of the phase 3 data, showing reductions in neutrophils, monocyte-derived macrophages, and inflammatory fibroblasts, along with increases in epithelial cells common to both treatments. Pseudo-time analyses identified four neutrophil subsets, transitioning from PADI4hi, OSMhi, and MX1hi to CXCR4hi populations. PADI4hi and OSMhi neutrophils exhibited high protease, cytokine (CXCL1, IL1B, OSM), and chemokine receptor (CXCR1, CXCR2) levels, while MX1hi expressed markers of IFN exposure. CXCR4hi neutrophils showed elevated CXCL2, TNF, and VEGFA levels. Notably, interactions between PADI4hi and OSMhi neutrophils and inflammatory fibroblasts, such as OSM and IL1B, were associated with MCS remission with both drugs. CXCR4hi neutrophils showed only minor changes unrelated to clinical outcomes. These findings suggest that neutrophils are highly heterogeneous, with abundant interactions in inflamed colonic tissue, potentially perpetuating chronic disease. Disrupting neutrophil interactions with myeloid and resident cells like inflammatory fibroblasts could reduce inflammation, possibly enhancing clinical remission rates.

## Introduction

Inflammatory bowel disease (IBD), including ulcerative colitis (UC), is a chronic inflammatory disorder of the gastrointestinal tract that has emerged as a globally relevant disease with increasing incidence worldwide and is associated with significant morbidity and healthcare utilization (1–3). Although biologic therapies with diverse mechanisms of action (e.g., anti-TNF, anti-integrin, anti-cytokines, JAK inhibitors) have expanded the therapeutic armamentarium of IBD, only approximately 30%–40% of patients respond to biologics, and a significant proportion lose response over time (4, 5). There remains an unmet clinical need to understand the mechanisms of biologic therapy failure in IBD. Identifying and targeting the specific cell types, cellular interactions, and cellular pathways that mediate response or resistance to biologics could lead to more effective and precise therapeutic strategies to improve clinical outcomes in patients with IBD.

Etrolizumab is a fully humanized monoclonal anti-integrin beta 7 (β7) antibody with a dual mechanism of action: blocking the homing of integrin α4β7-bearing lymphocytes to the gut and reducing the retention of αEβ7-bearing intraepithelial lymphocytes (6–8). The results from phase 3 (Ph3) studies of etrolizumab had mixed clinical outcomes (6–9). Among those, HIBISCUS I and II were two identical randomized, double-blind, placebo-controlled studies with head-to-head efficacy comparisons with adalimumab (anti-TNF) conducted in patients with moderately to severely active ulcerative colitis and naive to biologic treatment (9). In the HIBISCUS I study, etrolizumab showed a higher proportion of patients achieving remission during induction treatment relative to placebo; however, this effect was not replicated in HIBISCUS II. In prior studies, evidence suggested an impact on the frequency of αEβ7+ cells and reductions in infiltrating CD8+ T cells (6, 7). The complete effect of etrolizumab in the mucosa of UC patients has yet to be described. Given the mixed Ph3 clinical outcomes, it was of interest to understand changes in mucosal expression profiles of responder and non-responder patients with direct comparisons to an effective treatment such as adalimumab.

Several reports describe transcriptional programs associated with disease severity, response to treatment, and non-response to biologics (10–17). Both immune and non-immune cells have been implicated, and through the advancement of single-cell and spatial methods, the evidence is mounting, pointing to the important role of myeloid cells in disease and response (14, 18). It is widely appreciated in IBD that the presence and, importantly, the persistence of neutrophils are critical features linked to mucosal inflammation as well as a vital component found in Geboes, Nancy, and Robart’s histological indices used to grade histological disease severity or deep remission; however, datasets capturing neutrophils only recently emerged (18). Furthermore, neutrophil products such as fecal calprotectin have served as valuable biomarkers reflecting intestinal inflammation, and guidance suggests that combinations of symptoms and biomarkers could be used as a non-invasive tool to monitor endoscopic disease in patients (19).

We sought to characterize immune and non-immune populations, including inflammatory macrophages and neutrophils, and their interactions with resident cells to better characterize the relationship of changes in these populations within the etrolizumab Ph3 studies. The work herein comprehensively evaluates the transcriptional profiles of immune cells with single-cell granularity and characterizes their phenotype in moderate–severe UC patients with comparisons between matched uninflamed and inflamed tissue. Importantly, we assessed transcriptional changes within our etrolizumab- and adalimumab-treated UC patients within the HIBISCUS studies by leveraging UC single-cell datasets to deconvolve the on-treatment changes, especially within the myeloid compartment to assess cellular changes on treatment and relationships to response outcomes. Importantly, this study reports the most in-depth characterization of neutrophils in UC at the single-cell level and, through the application to clinical outcomes, provides a better understanding of the roles of neutrophils in response to treatment. In contrast to prior studies that linked myeloid gene signatures to treatment resistance, our study uniquely integrates phase 3 trial biopsies with a deeply annotated single-cell atlas of UC, enabling the first deconvolution of biologic response at the neutrophil subset level.

## Results

### Biologic therapies significantly change integrin gene expression and genes associated with inflammatory and epithelial cells

**Figure 1:**
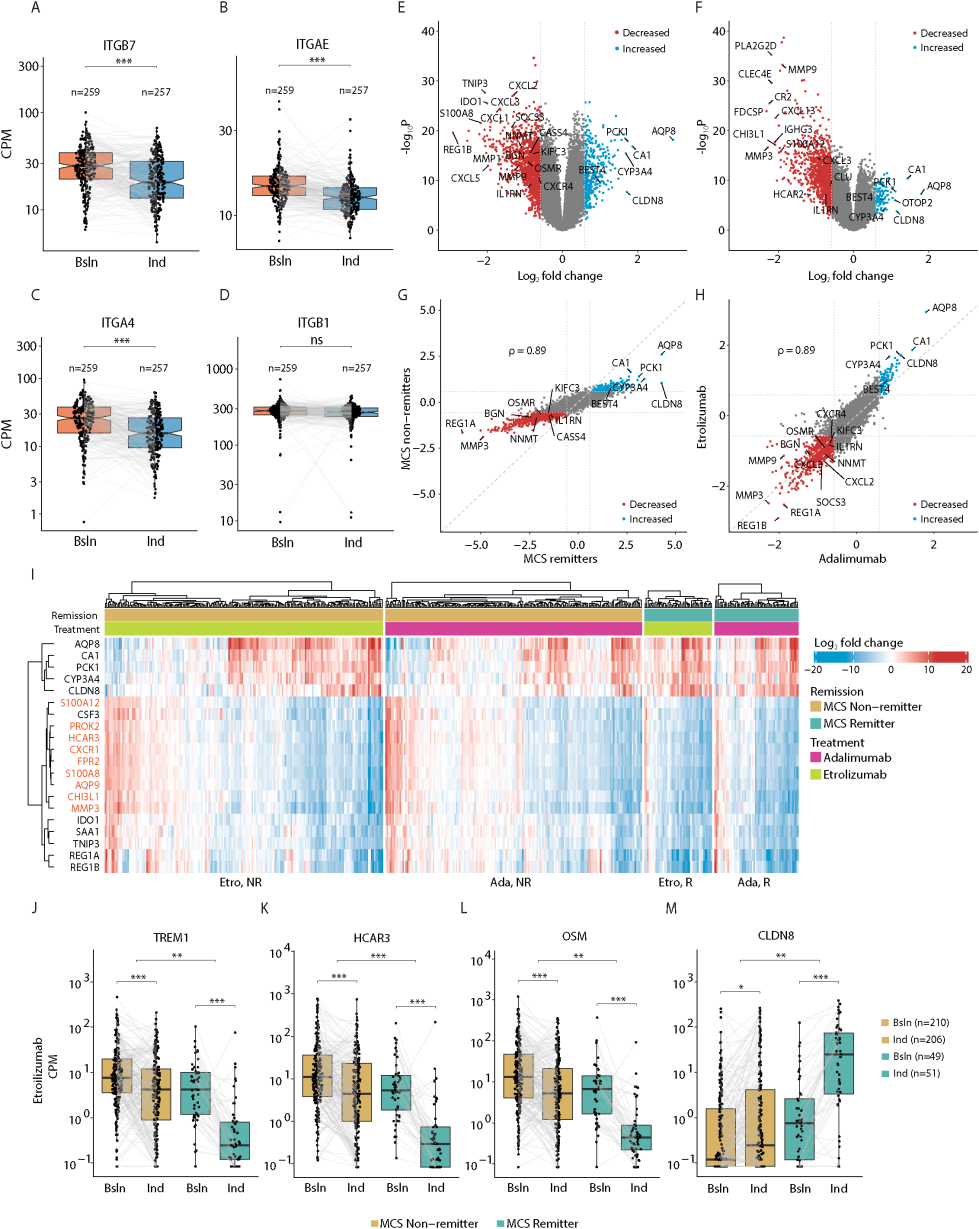
Transcriptomic analysis of etrolizumab, adalimumab, and placebo treatment in colonic biopsies. **(A–D)** Expression of selected integrins before (baseline) and after (induction; week 10) etrolizumab treatment. Each point represents an individual patient, and pre- and post-treatment trajectories for each patient are shown (gray bar). Expression values are in normalized counts per million (CPM). Box plots represent the upper and lower quartiles, the middle line represents the median, notches show 1.58× the interquartile range (IQR) divided by the square root of the number of samples measured, and the whiskers extend to the most extreme point not more than 1.5× outside of the interquartile ranges. **(E, F)** Volcano plots showing log2 fold change on the x-axis and -log10 *p*-value on the y-axis for changes in gene expression after etrolizumab **(E)** or adalimumab **(F)** treatment for 10 weeks. Each point represents a gene, colored blue for genes significantly upregulated after treatment (fold change >1.5× at an FDR of 0.05) and colored red for genes significantly downregulated after treatment (fold change <-1.5× at an FDR of 0.05). **(G)** Comparison of log2 fold changes after etrolizumab treatment in either remitters (x-axis) or non-remitters (y-axis). Each point represents a gene, colored as in **(E)** above. Spearman correlation coefficient comparing the log2 fold changes across the conditions is shown. **(H)** Comparison of log2 fold changes between adalimumab (x-axis) and etrolizumab (y-axis). Each point represents a gene, colored as in **(E)** above. Spearman correlation coefficient comparing the log2 fold changes across the conditions is shown. **(I)** Heatmap of log2 fold changes of top differentially expressed genes after etrolizumab treatment. Each row shows a gene, and each column shows the difference in expression for a patient between week 10 and baseline measures. Patients are grouped by treatment arm and remission status. Labels from genes previously reported to be expressed by myeloid cells are shown in orange. **(J–M)** Expression of selected neutrophil-associated or epithelium-associated genes before (baseline) or after (induction; week 10) etrolizumab treatment. Each point represents a sample collected from a patient at the respective time point, and patient measurements are joined by a line. Boxes are colored by the remission status of the patient at week 10. Boxes represent the upper and lower quartile, the middle line represents the median, and the whiskers extend to the most extreme point not more than 1.5× the interquartile range from the box boundaries. For all panels, stars indicate Benjamini–Hochberg corrected *p*-values: ns—*p* > 0.01; *p < 0.01; **p < 0.001; ***p < 0.0001.

We performed bulk RNA sequencing on colonic biopsies from the HIBISCUS I and II clinical trials to evaluate mechanistic and pathobiological changes in gene expression (**Supplementary Table S1**). Given etrolizumab's mechanism of action, integrin gene expression was assessed to infer changes in β7-expressing cells, including α4β7+ and αEβ7+ populations. Baseline integrin expression was similar across all treatment groups. Significant reductions in ITGA4 and ITGB7, but not ITGB1, were noted after etrolizumab and adalimumab treatment (**Figures 1A–D**; **Supplementary Figures S1A–D**). ITGAE expression decreased only with etrolizumab. Placebo treatment showed no significant integrin gene changes (**Supplementary Figures S1E–H**). The results of a transcriptome-wide analysis revealed that >1,800 genes changed significantly after etrolizumab (1,261 decreased, 347 increased) or adalimumab (1,137 decreased, 112 increased) treatment, with at least 1.5-fold change at a false discovery rate (FDR) of 0.05 (**Figures 1E, F**; **Supplementary Table S2**).

Gene expression changes post-treatment with etrolizumab were similar in MCS non-remitters and remitters, however these changes were more pronounced in remitters (**Figure 1G**). Changes were highly concordant between etrolizumab and adalimumab (Spearman rho = 0.89) (**Figure 1H**; **Supplementary Table S2**). Placebo-treated patients showed similar but less pronounced changes, with fewer significant gene changes (179 decreased, 43 increased, FDR of 0.05; **Supplementary Figures S1I–K**).

Most genes with decreased expression were linked to immune cells and inflammatory processes, while genes with increased expression were associated with epithelial cells (**Supplementary Table S3**). Examining differentially expressed genes (DEGs) with the highest changes, myeloid lineage cell genes, including neutrophils, were notably enriched (**Figure 1I**). Top DEGs like TREM1, HCAR3, and OSM were significantly downregulated after etrolizumab (**Figures 1J–L**) or adalimumab (**Supplementary Figures S1L–N**) treatment, with greater changes in remitters. Epithelial genes like CLDN8 increased after treatment, especially in remitters (**Figure 1M**; **Supplementary Figure S1O**). These trends were not significant in placebo-treated patients (**Supplementary Figure S1P–S**).

### Identification of immune and non-immune cell populations via the generation of an expanded UC single-cell RNA-Seq atlas

**Figure 2:**
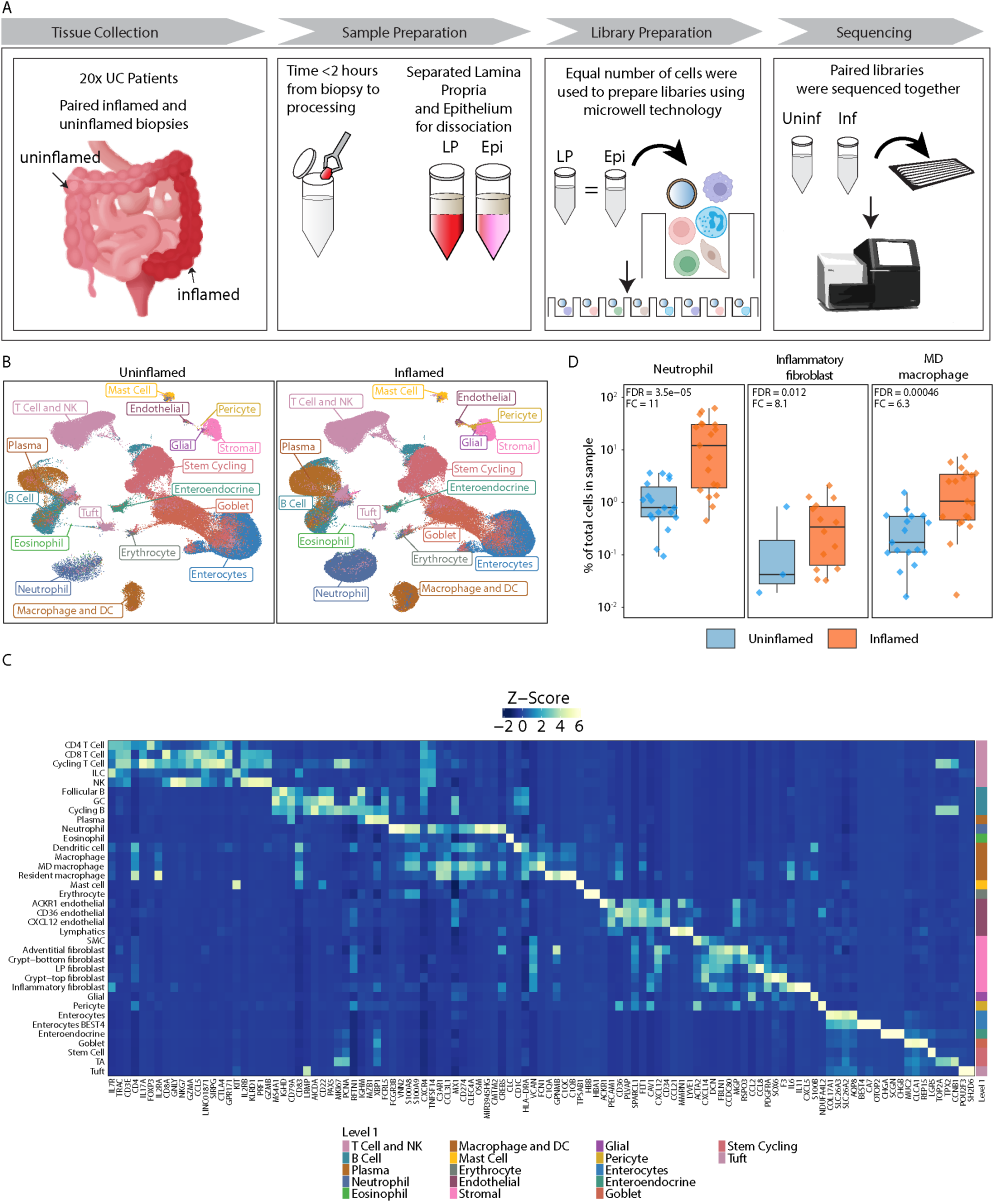
Generation of an ulcerative colitis single-cell atlas that captures tissue neutrophils. **(A)** Experimental design. Biopsies were collected from both inflamed and uninflamed tissue from ulcerative colitis patients. To retain sensitive cells, including neutrophils, sample processing time was <2 h from biopsy collection. We used the Rhapsody microwell platform to collect whole transcriptome data. **(B)** UMAP of cells annotated at the broadest classification level. Each point represents an individual cell derived from uninflamed tissues (left panel) or inflamed colonic tissue (right panel), with each cell population colored by the lineage. **(C)** Relative abundance of differentially abundant lineages. Relative abundance is shown as the percentage of total cells in each sample for neutrophils, inflammatory fibroblasts, and monocyte-derived macrophages across inflamed and uninflamed samples (neutrophil: *n* = 18 uninflamed, 19 inflamed; inflammatory fibroblast: *n* = 3 uninflamed, 14 inflamed; monocyte-derived macrophage: *n* = 17 uninflamed, 19 inflamed). Fold difference and FDR values were calculated as described in “Materials and methods”. FDR values were considered significant if they were <0.05. **(D)** Heatmap of marker genes for intermediate-level annotations. Columns show individual genes specific to an individual cell type or broader lineage. Colors represent the centered and scaled pseudo-bulk expression levels. Broad lineage classification is demonstrated by the color bar on the right.

Given the high proportion of myeloid-derived genes differentially expressed in bulk RNA sequencing from colonic tissue and their association with disease activity, further analyses were conducted to characterize sub-populations within major cell types and understand gene expression changes related to disease activity. We collected biopsies from inflamed and uninflamed colonic tissue from 20 moderate to severe UC patients, including 18 matched pairs (**Figure 2A**, **Supplementary Table S4**). In total, data from 408,930 cells were generated: 236,855 from inflamed tissue and 172,075 from uninflamed tissue. Using a semi-automated annotation approach (see “Materials and methods”), 17 major cell populations were identified, including stromal, epithelial, adaptive, and innate immune cells, with 47,516 neutrophils (**Figure 2B**; **Supplementary Figure S2A**). Increased inflammatory cells, including neutrophils, were observed in biopsies from inflamed areas with high endoscopic scores (**Supplementary Figure S2B**).

Detailed annotation identified 36 cell subsets including T cell subsets (CD4+ T helper, CD8+ cytotoxic, KI67+ cycling T cells), B cell subsets (IGHD+FCER2+ follicular, MS4A1+AICDA+MKI67- germinal center, MKI67+PCNA+ cycling, MZB1+XBP1+PRDM1+ plasma cells/plasmablasts), myeloid subsets (VCAN+ monocyte-derived macrophages (MD macrophage), C1QA+ resident macrophages, CD1C+ dendritic cells, VNN2+ neutrophils, KIT+ mast cells, CLC+ eosinophils), IL7+ innate lymphoid cells (ILCs), NKG7+ natural killer (NK) cells, endothelial cells (ACKR1+, CD36+, CXCL12+ subsets), S100B+ glial cells, RGS5+ pericytes, HBB+ erythrocytes, epithelial cells (CA1+CA2+ enterocytes, BEST4+ enterocytes, CHGA+ enteroendocrine, MUC2+ goblet, SH2D6+ tuft cells, PCNA+LGR5+ stem cells, TOP2A+ transit amplifying), ACTA2+ smooth muscle cells (SMC), and fibroblasts (PI6+ adventitial, WNT2B+ crypt-bottom, PDGFRA+ crypt-top, ADAMDEC1+ABCA8+ lamina propria, IL11+ inflammatory) (**Figure 2C**; **Supplementary Figure S3A**, **Supplementary Table S5**).

This single-cell atlas identified genes associated with myeloid lineages, including macrophages and neutrophils, which have been previously linked to resistance to biologic therapies (**Figure 1G**) (14, 20, 21). Significant differences in cell subset abundance (negative binomial test, FDR < 0.05) showed elevations in neutrophil, MD macrophage, inflammatory fibroblast, pericyte, and ACKR1+ endothelial cell levels and reductions in BEST4+ enterocyte and adventitial fibroblast levels (**Figure 2D**; **Supplementary Figures S2C, D**).

### Cellular context of gene expression changes with disease and treatment

**Figure 3:**
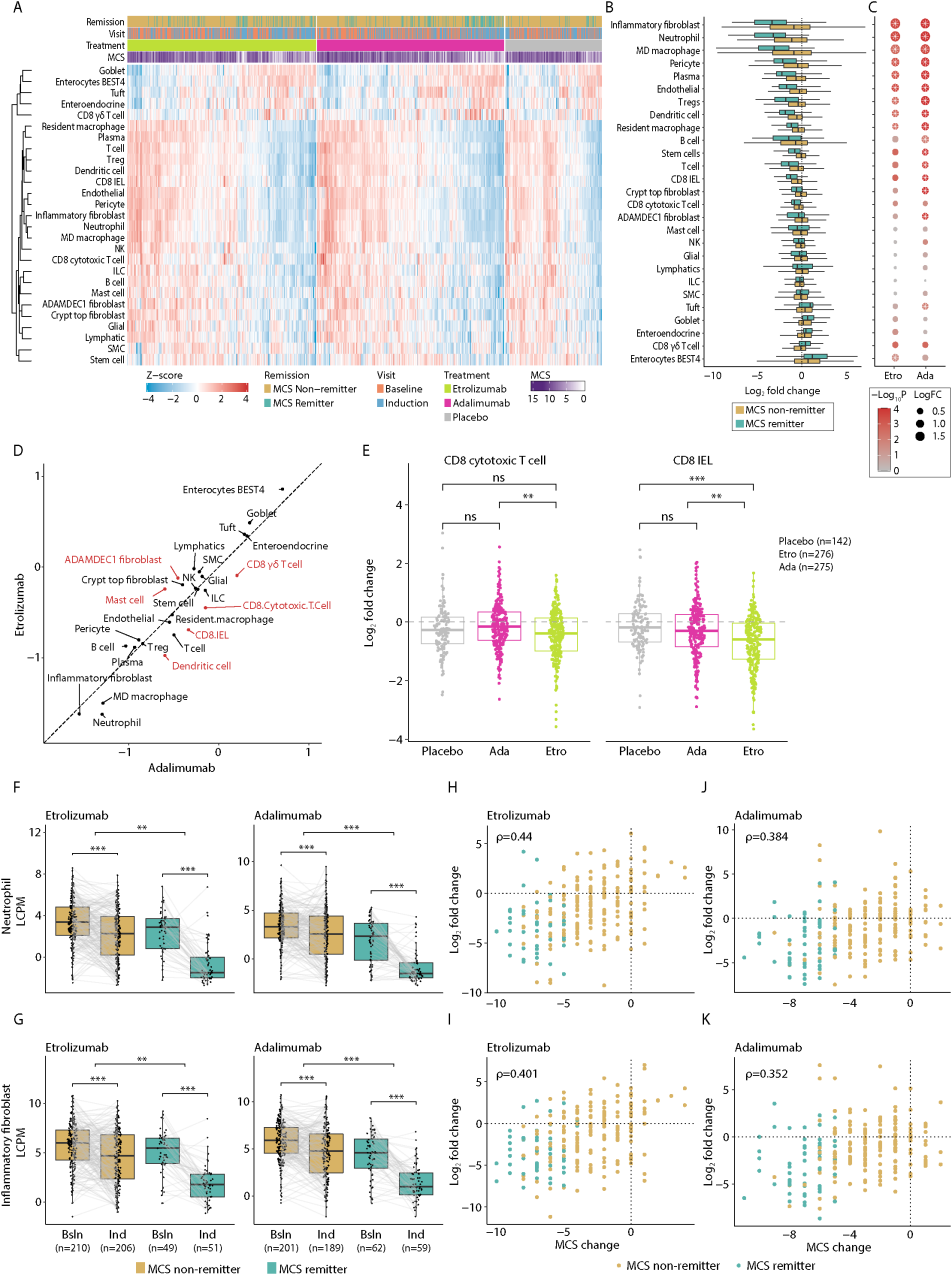
Neutrophils, monocyte-derived macrophages, and inflammatory fibroblasts are reduced by treatment, especially in remitters. **(A)** Heatmap of signature scores for lineages defined in the single-cell atlas. Each row shows centered and scaled signature scores for a given population in individual samples from patients before or after treatment with etrolizumab, adalimumab, or placebo as indicated. **(B)** Effect of treatment on genes specific for individual cell populations in remitters or non-remitters after etrolizumab treatment (left panel). Boxes represent the log2 fold change between baseline and week 10 for either non-remitters or remitters. Cell populations are sorted by the magnitude of difference between longitudinal log2 fold change in remitters vs. non-remitters. **(C)** Significance of difference in log2 fold change between remitters and non-remitters after either etrolizumab or adalimumab treatment. Each point is colored by the *p*-value from comparing the effect in remitters and non-remitters, with the size showing the log2 difference between the fold changes. White stars indicate a >1.5-fold statistically significant difference in remitter vs. non-remitter response at an FDR of 0.05. **(D)** Effect of adalimumab (x-axis) or etrolizumab (y-axis) on individual cell type expression signatures. Each point represents an individual cell population, colored by those significantly different between etrolizumab and adalimumab (red) or not (black). **(E)** Log2 fold changes of selected T cell populations after placebo, adalimumab, or etrolizumab treatment. Each point represents the difference between week 10 and baseline measurements of a cell type for either CD8+ IELs (left) or CD8+ cytotoxic T cells (right). Stars indicate nominal Wilcoxon signed-rank test *p*-values: ns—*p* > 0.01; *p < 0.01; **p < 0.001; ***p < 0.0001. **(F, G)** Expression of neutrophil **(F)** or inflammatory fibroblast **(G)** signature scores before and after treatment with either etrolizumab or adalimumab. Each point represents a patient sample before (baseline) or after (induction, week 10) treatment. Samples collected from the same patient are linked by a line. **(H, I)** Comparison of log2 fold change in neutrophil **(H)** or inflammatory fibroblast **(I)** gene expression signature (y-axis) with change in Mayo Clinic score (x-axis) after treatment with etrolizumab. Each point represents an individual patient, colored by remission status at week 10. **(J, K)** Comparison of log2 fold change in neutrophil **(J)** or inflammatory fibroblast **(K)** gene expression signature (y-axis) with change in Mayo Clinic score (x-axis) after treatment with adalimumab. Each point represents an individual patient, colored as in **(H)**. For **(F)** and **(G)**, stars indicate Benjamini–Hochberg corrected *p*-values: ns—*p* > 0.01; *p < 0.01; **p < 0.001; ***p < 0.0001.

We utilized this single-cell atlas to create cell-specific gene modules to deconvolve the bulk transcriptomic data from the HIBISCUS studies, thereby elucidating cellular changes in etrolizumab- and adalimumab-treated patients. Each gene module was tailored to specific cell populations and sub-populations within related cellular lineages (see “Materials and methods”) (**Supplementary Figure S3A**; **Supplementary Table S6**). Our analyses revealed a coordinated expression across most immune and stromal populations and opposite expression patterns in epithelial cell subsets, regardless of treatment (**Figure 3A**; **Supplementary Figure S4A**). Higher expression markers of immune cells (e.g., macrophages, neutrophils, T cells) and stromal populations (e.g., pericytes, fibroblast subsets, glial cells) were associated with higher MCS scores, irrespective of sampling time. Conversely, a high expression of genes primarily found in epithelial cell populations, including goblet cells, BEST4+ enterocytes, enteroendocrine cells, and tuft cells, was associated with lower MCS scores (**Figure 3A**).

Upon comparing changes in cell-type-specific modules in remitters and non-remitters after 10 weeks of induction treatment with etrolizumab, it was demonstrated that the largest differences were found in neutrophils, MD macrophages, and inflammatory fibroblasts (**Figure 3B**). Consistent with these findings, comparing inflamed and uninflamed samples in the single cell dataset, a significant increase in genes associated with BEST4+ enterocytes was observed after treatment, with more substantial increases found in remitters than non-remitters. The average change in cell-type-specific genes in patients treated with etrolizumab was inversely correlated with the differential abundance of these cell populations in inflamed vs. uninflamed biopsies (Spearman correlation = -0.86, **Supplementary Figure S4B**). Changes post-treatment appear largely treatment agnostic, with most populations showing a similar behavior between etrolizumab and adalimumab treatment arms (**Figures 3B, C**; **Supplementary Figure S4C**). Notable exceptions include CD8+ T lymphocyte subsets, including intra-epithelial lymphocytes (IELs), CD8+ cytotoxic T cells and γδT cells, and dendritic cells, which were reduced to a greater extent following etrolizumab treatment, and ADAMDEC1+ fibroblasts and mast cells, which were reduced more following adalimumab treatment (**Figures 3D, E**). The cell specificity of the integrins was also observed to vary, with ITGAE, ITGB7, and ITGA4 showing higher specificity for T cell subsets, while ITGB1 was expressed on many stromal cells. ITGB7 and ITGA4 were additionally expressed on dendritic cells, resident macrophages, and plasma and B cell subsets (**Supplementary Figure S4D**). Upon comparing relative changes across neutrophil and inflammatory fibroblast-associated genes, greater reductions were observed in patients who achieved MCS remission (**Figures 3F, G**). Furthermore, reductions in neutrophil and inflammatory fibroblast-associated genes were correlated with overall changes in MCS irrespective of remission status or biologic treatment (**Figures 3H–K**; **Supplementary Figures S4E–H**, Spearman rho = 0.44, 95% CI: 0.32–0.55 for neutrophils, rho = 0.40, 95% CI: 0.28–0.51 for inflammatory fibroblasts after etrolizumab treatment and rho = 0.38, 95% CI: 0.26–0.49 for neutrophils, rho = 0.35, 95% CI: 0.23–0.47 for inflammatory fibroblasts after adalimumab treatment). Supporting the role of neutrophil subsets and inflammatory fibroblast as crucial cell types for MCS remission, previous anti-TNF resistance modules were expressed at the highest levels by both cell types (**Supplementary Figure S5A**) (12).

### Heterogeneity of neutrophils in the colonic tissue of UC patients

**Figure 4:**
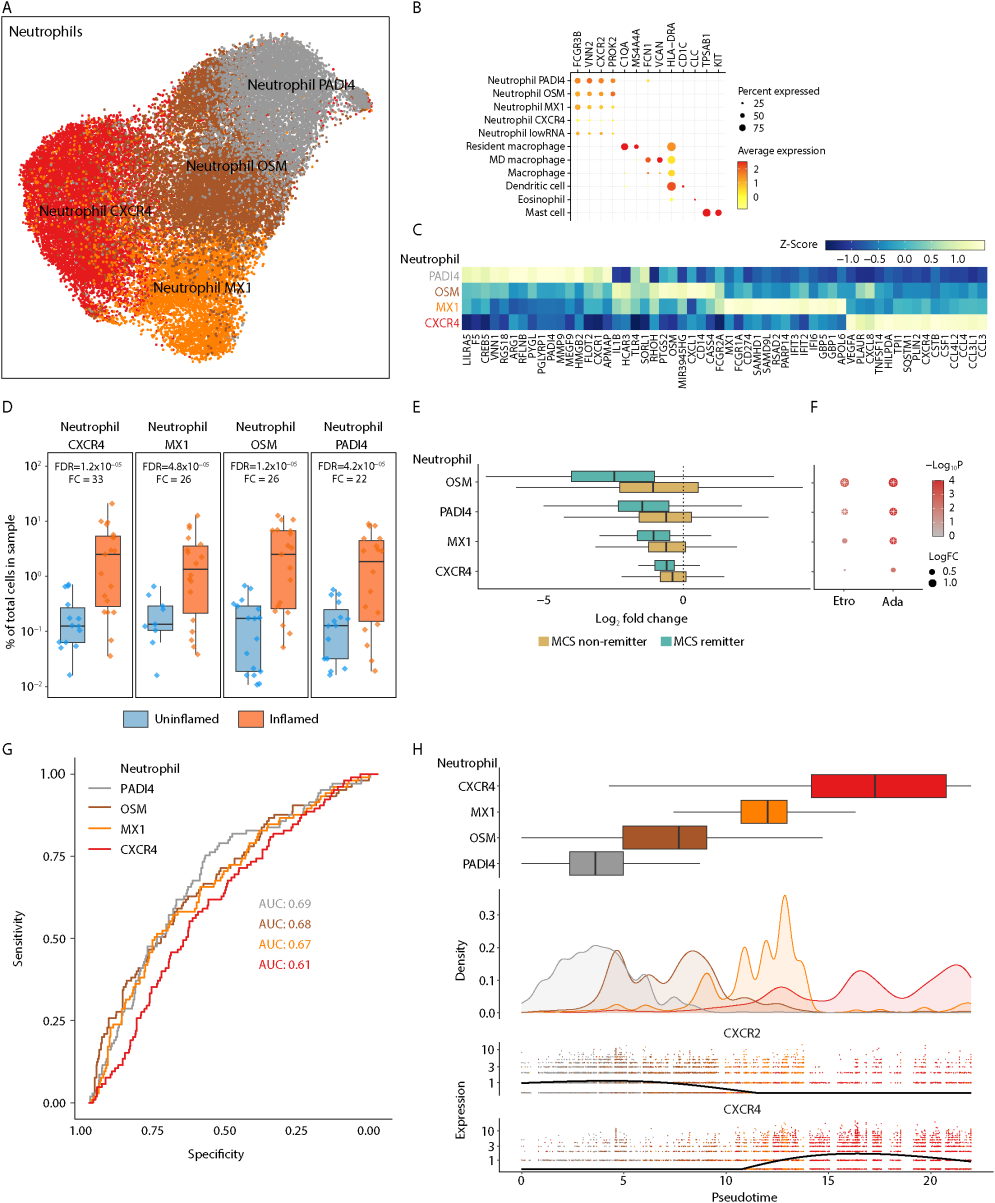
Heterogeneity of neutrophils in UC tissue biopsies. **(A)** UMAP projection of neutrophil subsets. Each point represents a cell, colored by the respective subset it was classified as. **(B)** Dotplot of marker genes for myeloid subsets. Each point represents the expression of a gene in a cell population. The size of the point indicates the proportion of cells expressing the gene, and the color indicates the centered and scaled expression level. **(C)** Heat map of marker genes for each neutrophil subset. Colors show centered and scaled gene expression values across four subsets of neutrophils. **(D)** Differential abundance of neutrophils across inflamed and uninflamed tissue. Each point represents a sample. The y-axis shows the fraction of each neutrophil subset relative to the total number of cells in that sample. Fold changes and FDR values were determined as described in “Materials and methods”. FDR values were considered significant if they were <0.05. Boxes represent the lower and upper quartiles, with the center line representing the median. Whiskers extend to the most extreme point no more than 1.5× the IQR from the box boundaries. **(E)** Log2 fold changes of gene expression signatures from each neutrophil subset after etrolizumab treatment, split by remission status at week 10. Cell populations are sorted by the magnitude of difference between longitudinal log2 fold change in remitters vs. non-remitters. **(F)** Significance of difference in log2 fold change between remitters and non-remitters after either etrolizumab or adalimumab treatment. Each point is colored by the *p*-value from comparing the effect in remitters and non-remitters, with the size showing the log2 difference between the fold changes. White stars indicate a >1.5-fold statistically significant difference in remitter vs. non-remitter response at an FDR of 0.05. **(G)** ROC curves for the association of longitudinal changes in neutrophil subsets with remission. **(H)** Pseudotime inference for neutrophil subsets. The x-axis represents the ordering of cells through pseudotime. Boxplots show the relative placement of neutrophil subsets across pseudotime, with density plots showing the total distribution of cells. Normalized expression values for CXCR2 or CXCR4 are shown across pseudotime, with the y-axis representing log2 normalized counts and each point representing a cell positioned in pseudotime and colored by the subset it belongs to.

Given the notable increase in neutrophils within inflamed tissue compared to uninflamed tissue and their association with persistent disease despite etrolizumab and adalimumab treatment, we aimed to characterize these cells further to understand their heterogeneity in inflammatory conditions such as UC. Four major neutrophil subsets were identified in both inflamed and uninflamed biopsies, along with one subset that could not be further classified due to low sequencing depth (**Figure 4A**; **Supplementary Figure S6A**). All subsets expressed neutrophil lineage markers, including FCGR3B, VNN2, CXCR2, and PROK2, while lacking markers of closely related myeloid cells, such as VCAN and CD300E in MD macrophages, HLA-DR in macrophages and dendritic cells, and CLC in eosinophils (**Figure 4B**). Specific markers such as CXCR4, OSM, MX1, and PADI4 were used to further classify these neutrophil populations. MX1hi neutrophils co-expressed several markers consistent with interferon signaling, including MX1 itself, IFI6, IFIT2, and IFIT3 (**Figure 4C**). OSMhi neutrophils expressed high levels of inflammatory cytokines and chemokines, including OSM, IL1B, and CXCL1 (**Figure 4C**; **Supplementary Table S7**). PADI4hi neutrophils expressed high levels of several membrane-bound proteases, including MMP9, MMP25, and MME (**Figure 4C**, **Supplementary Table S7**). Lastly, CXCR4hi neutrophils produced a unique set of cytokines: CCL3, CCL4, VEGFA, and CSF1 (**Figure 4C**). All four subsets were significantly more abundant in inflamed colonic tissue compared to uninflamed tissue (**Figure 4D**; **Supplementary Figure S6B**). A smaller dataset of neutrophils from colonic biopsies identified three subsets (N1, N2, and N3), which exhibited similar expression patterns to PADI4/OSM, CXCR4, and MX1 neutrophils, respectively (**Supplementary Figure S6C**) (18).

Comparing gene sets representative of these four neutrophil subsets in the HIBISCUS bulk RNA sequencing data, the most extensive changes post-treatment were observed in genes expressed by OSMhi and PADI4hi neutrophil populations, with significant differences between remitters and non-remitters (**Figure 4E**). The reductions observed were treatment-agnostic, showing the same pattern after etrolizumab and adalimumab treatment (**Supplementary Figure S6D**; **Supplementary Table S8**; **Figure 4F**). The marked reduction of PADI4hi- and OSMhi-specific genes was more closely associated with remission, suggesting that these subsets were related to residual disease (**Figure 4G**). The pre-treatment levels of these gene modules showed modest prognostic capacity, suggesting that, with further validation, PADI4hi/OSMhi signatures could serve as biomarkers to stratify UC patients at risk of biologic non-response. Prospective validation in independent cohorts will be essential (**Supplementary Figure S6E**).

Previous reports indicate that neutrophils regulate CXCR2 and CXCR4 expression throughout their life cycle (22). Consistent with this, a gradient of CXCR2 and CXCR4 expression was observed across the four neutrophil subsets, suggesting that they represent points along a differentiation trajectory (**Supplementary Figure S6F**). Pseudotime inference created a trajectory through the neutrophil single-cell data, showing a gradient of decreasing CXCR2 and increasing CXCR4 expression, respectively, over pseudotime (**Figure 4H**). The trajectory ran from PADI4hi through OSMhi and MX1hi, ending with the CXCR4hi state (**Figure 4H**). Consistent with this model, a subpopulation of MMP9 and bactericidal-permeability-inducing protein (BPI) expressing cells within the PADI4hi subset suggested the presence of immature neutrophils (**Supplementary Figure S6G**) (23, 24). More mature neutrophils in the inferred pseudotime trajectory expressed cytokines/chemokines such as CCL3, CCL4, and VEGFA (**Supplementary Table S9**; **Supplementary Figure S7A**).

### Rewiring of cell–cell communication in neutrophil subsets in the inflamed tissue

**Figure 5:**
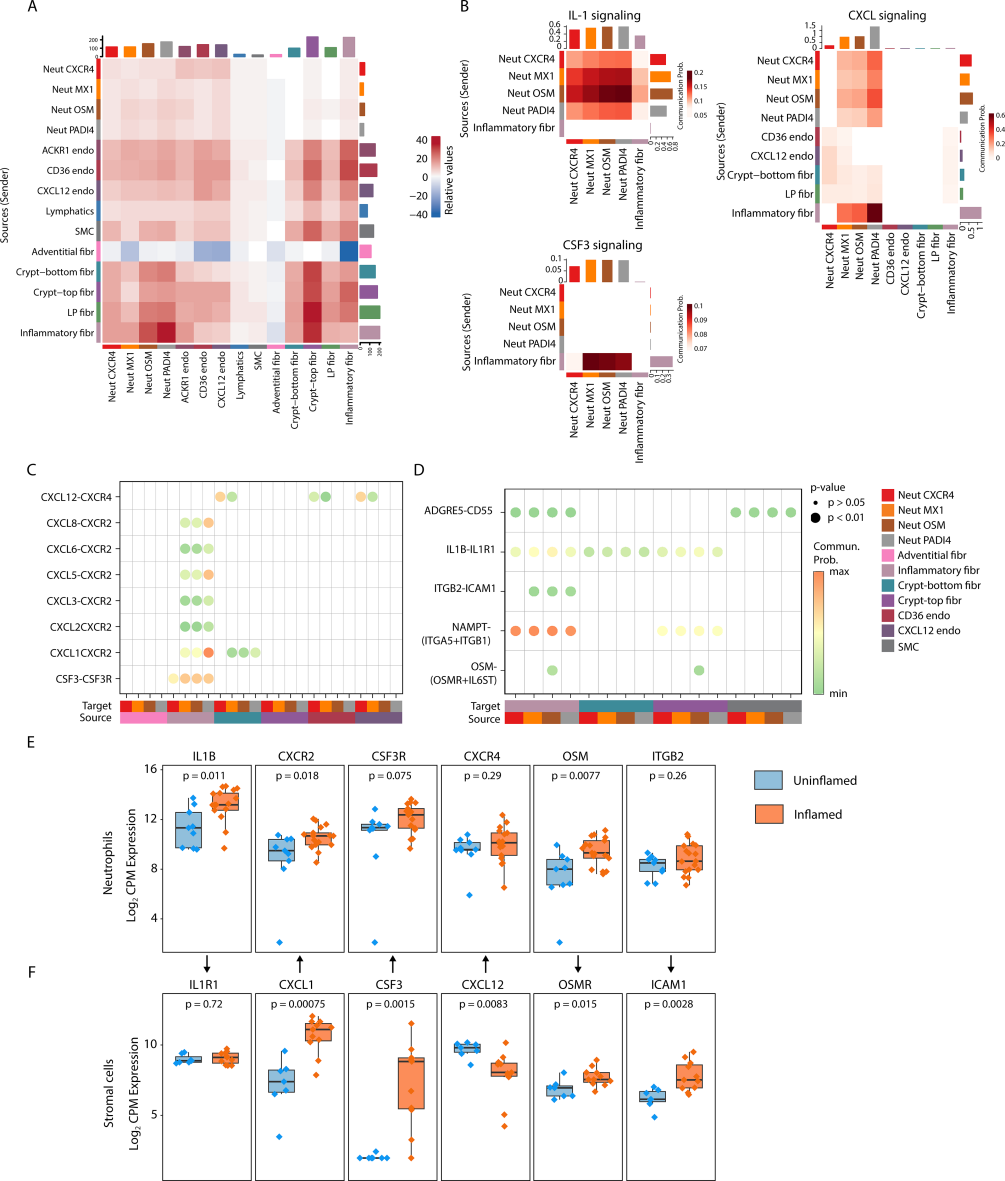
Neutrophil interaction rewiring in UC inflamed tissue. **(A)** Heatmap of the differential number of inferred interactions between inflamed and uninflamed tissue among neutrophil and stromal subpopulations. **(B)** Heatmap of signaling pathways CXCL, CSF3, and IL1 showing significant communications between neutrophil subpopulations and inflammatory fibroblasts in inflamed tissue. Significant interactions were identified using a permutation test and were considered significant if the null distribution was significantly different than the observed distribution with a *p*-value <0.05. The color in the heatmap is proportional to the overall communication probability between each cell type. **(C)** Selected significant ligand–receptors as incoming signaling interactions to neutrophil subpopulations from fibroblasts and endothelial subsets in the inflamed tissue. **(D)** Significant ligand–receptors were selected as outgoing signaling interactions between neutrophil subpopulations, fibroblasts, and endothelial subsets in the inflamed tissue. **(E)** Pseudo-bulk expression levels of ligands and receptors in the neutrophil population. Expression values are shown on the y-axis as log2 normalized counts per million (CPM). **(F)** Pseudo-bulk expression levels of ligands and receptors in the stromal population. Expression values are in log2 normalized counts per million (CPM). Based on the Wilcoxon test, *P*-values are reported for significant change in expression between the inflamed (orange) and uninflamed (blue) tissue.

Given the increased abundance of neutrophil subsets in inflamed tissue and previously described association with inflammatory fibroblasts (12), elevations in inflamed tissue, and modulation by etrolizumab and adalimumab, we analyzed cell–cell communication networks to characterize predicted interactions between neutrophil states and other cells. The most substantial predicted ligand–receptor interactions were between all neutrophil subsets with multiple stromal populations, especially in inflamed tissues compared to uninflamed tissue, with both PADI4hi and OSMhi neutrophil subsets showing the most significant difference in interaction potential (**Figure 5A**; **Supplementary Figure S8A**). Meaningful interactions within signaling pathways from inflammatory fibroblasts to all neutrophil subsets were also identified (**Figure 5B**; **Supplementary Figure S8B**). Chemokine interactions were abundant from inflammatory fibroblasts to PADI4hi, OSMhi, and MX1hi neutrophil subsets via CXCL1/2/3/5/6/8 expressed by fibroblasts and CXCR1/2 expressed on neutrophils (**Figure 5C**). In addition, interactions were predicted between crypt-bottom fibroblasts, CD36 endothelial cells, and CXCL12+ endothelial cells with CXCR4hi and MX1hi neutrophils via CXCL12 expressed by stromal cells and CXCR4 expressed by neutrophils (**Figure 5C**). Among all ligand–receptor interactions, only CXCR4:CXCL12 interactions were present between these stromal cells and CXCR4hi neutrophils in uninflamed tissue, as were CXCL1:CXCR2 interactions between inflammatory fibroblasts and PADI4hi, OSMhi, and MX1hi neutrophils, albeit in both cases the signaling strength was reduced in uninflamed tissue (**Supplementary Figures S8B, C**).

Significant IL-1 signaling was inferred across all neutrophil populations to inflammatory fibroblasts, with more robust signaling from PADI4hi and OSMhi neutrophils (**Figures 1E**, **5B, D**). This interaction was also suggested within uninflamed colonic tissues (**Supplementary Figures S8B, D**). Conversely, we predict CSF3 signaling from inflammatory fibroblasts with all neutrophil populations through CSF3:CSF3R (**Figures 5B, C**). OSM: OSMR signaling was also identified as a strong interaction between neutrophils, specific to OSMhi neutrophils, and inflammatory and crypt-top fibroblasts (**Figure 5D**). Beyond cytokines and chemokines, other notable interactions were inferred to be acting on receptors on inflammatory fibroblasts, including NAMPT : ITGA5/ITGB1, ITGB2:ICAM, and ADGRE5:CD55. The increased expression of receptors and ligands specific to the neutrophils and fibroblast populations between inflamed and uninflamed tissue supports the predicted increased crosstalk between these populations (**Figures 5E, F**).

## Discussion

UC is an immune-mediated inflammatory disease driven by genetic predisposition, environmental triggers, and dysregulation of immunity, leading to damage to the intestinal barrier (1–3). Key immune infiltrates include T and B lymphocytes, plasmablasts, and myeloid populations (14–16)—for example, IL17-expressing T cell subsets are reported to expand in the inflamed gut of UC patients, contributing to the TH17-driven pathobiology and tissue damage (16). The GIMATS module reported by Martin et al., comprising activated dendritic cells, inflammatory macrophages, activated T cells, IgG plasma cells, activated fibroblasts, and endothelial cells, and myeloid infiltration into the colonic epithelium reported by Jha et al. were shown to be associated with non-response to anti-TNFs and more severe diseases (14, 15). These cells mediate cytotoxicity against epithelium, aberrant B cell-mediated antibody production, regulatory T cell resistance, and neutrophil-driven ulceration (14, 25–31). In the non-immune compartment, it has been reported that a greater abundance of inflammatory fibroblasts may sustain the continued recruitment of immune cell populations such as myeloid cells via cytokine/chemokine signaling (12, 15, 17). Breaking this cycle of inflammation has been successful in some patient populations with anti-TNFs, anti-integrins, JAK inhibitors, and, more recently, IL-23 and TL1A inhibitors. However, patterns of non-response across these agents appear consistent in that the presence of inflammatory cells and inflammatory pathways is not fully normalized relative to patients in remission (10–17, 32, 33).

The etrolizumab phase 3 studies included well-characterized patients and biomarker assessments, enabling rigorous molecular evaluations of colonic tissues before and after treatment. This report shares results from pooled HIBISCUS I and II studies, which were identical placebo-controlled studies that compared the clinical efficacy of etrolizumab and adalimumab in treatment-naive UC patients.

Overall, we observed significant reductions in genes expressed by memory T and B cell subsets, including genes that encode alpha 4, alpha E, and beta 7 integrins that form heterodimeric complexes and allow for mucosal tissue homing. Despite the significant reductions in ITGA4 and ITGB7, there was minimal to no change in ITGB1, potentially due to expression in other cellular populations (**Supplementary Figure S4D**). Similar observations were made in post-treatment biopsies from a phase 3 etrolizumab study in Crohn’s disease (7). Although these findings align with the proposed dual action of etrolizumab, similar results were seen with adalimumab treatment, suggesting that the changes are due to reduced inflammatory infiltrate following a decrease in disease severity. A unique pharmacodynamic effect of etrolizumab was the reduced expression of ITGAE- and IEL-specific genes, implying decreased retention or increased egress of αEβ7+ lymphocytes. This insight aligns with findings from the EUCALYPTUS and *in vivo* studies (6, 7). Analyzing colonic samples earlier in the treatment may provide clearer insights into these effects.

Both etrolizumab- and adalimumab-induced significant transcriptional changes, decreasing inflammation-associated genes, and slightly increasing epithelial-cell-related genes. The degree of these alterations correlated with changes in MCS or remission status. Notably, alterations in myeloid lineage and inflammatory fibroblast gene expression were closely associated with MCS changes. Our data confirmed previous reports linking non-response to TNF blockers with myeloid-expressed genes, such as OSM and IL1B, and extended this association to anti-integrins (**Supplementary Figure S5A**) (12, 17, 34, 35). This suggests that these changes reflect disease state or resolution rather than specific mechanisms. As a result, the concept of UC disease resolution may expand to include the restoration of molecular pathways altered in disease pathogenesis (36, 37).

Despite the relevance of myeloid cells, single-cell data on specific subsets, particularly neutrophils, have been limited due to technical challenges associated with cell viability and low transcript levels. Single-cell RNA sequencing of over 400K cells identified distinct myeloid populations, including >30K neutrophils. As expected, we observed an increased abundance of neutrophils, MD macrophages, and inflammatory fibroblasts in inflamed tissue, along with a significant reduction in BEST4+ enterocytes. The bulk transcriptomic data from the HIBISCUS trials also reflected this inverse relationship between immune and epithelial cells. Single-cell analysis, applied to the HIBISCUS dataset, confirmed significant reductions in neutrophils, MD macrophages, and fibroblasts with both etrolizumab and adalimumab, reinforcing the role of these cells in UC pathobiology. Disruptions were also evident in the epithelial layer, involving resident γδT cells, TH17 cells, and inflammatory myeloid cells (14).

Four sub-populations of neutrophils distinguished by CXCR4, OSM, MX1, and PADI4 expression exhibited unique gene profiles. The diversity of expressed genes across neutrophil subsets highlights their varied roles. A low RNA abundance neutrophil subset was observed but excluded from analysis due to uncertain biological relevance. All four neutrophil subsets were present in both inflamed and uninflamed biopsies. In HIBISCUS, reductions in OSMhi and PADI4hi subsets were linked to remission, while CXCR4hi neutrophils showed no such association.

Pseudotime analysis suggested a differentiation trajectory for neutrophils, from immature to mature, along the CXCR2 axis (22, 38, 39). Rather than discrete subsets, our data support a model where these states exist along a maturation continuum, with PADI4hi neutrophils representing an early, proteolytic-primed state and CXCR4hi neutrophils a tissue-resident, possibly terminal state. Neutrophil gene expression in UC aligns with previous studies on cell state trajectories in rodents (39). Consistent with this model, we found the highest expression of immature neutrophil markers, including MMP9 (gelatinase B) and BPI in PADI4hi cells, with reduced expression along our pseudotime trajectory (23, 24).

Cell state differences suggest functional variations inferred through ligand–receptor interactions between neutrophils and resident cells. OSMhi and PADI4hi neutrophils interacted with fibroblasts via enriched OSM and IL1B pathways. Friedrich et al. also identified IL-1b-driven interactions between neutrophils and fibroblast subsets, independent of TNF-a, suggesting a key mechanism for resistance to anti-TNF therapies (12). In addition, we found that CXCL12 signaling between fibroblasts and mature neutrophils persisted in all conditions. Stromal inflammatory fibroblasts produce CXCL1/2/3/8, key ligands associated with neutrophil homing, through their interaction with CXCR1/2. Together with the expression of CSF3 as a crucial regulator of neutrophil production and increased lifespan, the data highlights a potential role for neutrophils’ immune response organization via cytokine signaling in the stromal compartment. Previous reports highlighted the role of CXCR4:CXCL12 signaling in IBD through homing of peripheral blood and lamina propria T cells, migration of B cells from Peyer’s patches, and a role for CXCL12 in both the inflamed state and homeostasis (40–42). How each of these immune cell compartments responds to CXCL12 signaling and under what conditions bears further study. The relevance of CXCR4hi neutrophils in IBD remains unclear, as they show less disease association than immature populations. CXCR4 has been described as a mechanism that facilitates the return of aging neutrophils to the bone marrow and retention within inflamed tissues, which may be reflected in this dataset and others. These findings highlight a potential role for neutrophils in organizing the immune response via cytokine signaling in the stromal compartment.

Neutrophils, their interactions and inflammatory products, may be key therapeutic targets in IBD, given their established role in chronicity of disease and non-response (13, 43). CXCR2 blockade in mouse models reduced neutrophil function, cytokine production, and pathology (44–46). While CXCR2 and IL-1b inhibitors have been tested in other diseases, limited data exist on their use in IBD, except in early-onset cases (47–49). Although neutrophils drive UC pathology, they are essential for mucosal defense. Therefore, direct targeting of neutrophils poses risks, especially in chronic diseases, due to potential immune compromise. These findings suggest that neutrophils may interact with resident cells in the inflamed gut. Additional experiments are required to validate these interactions and their role in mucosal immunity. Targeting neutrophil interactions could be a new therapeutic rationale—for example, by disrupting cell–cell communication or neutrophil activation in moderate to severe UC. Given the central role of PADI4hi and OSMhi neutrophils in sustaining inflammation through stromal interactions, therapeutic strategies that disrupt OSM: OSMR or IL1B:IL1R signaling, rather than broad myeloid suppression, may selectively dampen pathologic circuits while preserving mucosal host defense.

One caveat to these results is that most of these inferences are drawn from transcriptomic data. This is particularly the case for inferring ligand–receptor interactions and their downstream signaling pathways. Future studies focused on proteomic analyses or functional readouts would be an important step forward in characterizing the nature of these complex multicellular interactions and elucidating their role in disease biology.

## Materials and methods

### Clinical trial bulk RNA-sequencing

#### Study design and patients

Available colonic biopsies were assessed from TNF inhibitor-naive adult patients with moderate-to-severe UC enrolled in two identically designed, randomized, double-blind, placebo-controlled phase 3 studies, Hibiscus I and Hibiscus II (9). Additional study and patient details can be found in the published clinical report (9).

#### Bulk RNA biopsy collection and processing

Colonic biopsy pairs were collected from the most inflamed region (20–40 cm from the anal verge) of patients undergoing a colonoscopy procedure, and biopsies were immediately stored in RNAlater solution (cat. #AM7022, Invitrogen). Tissue pinch biopsies from trial subjects were stored in RNAlater (Qiagen) tubes, thawed, and placed into individual wells of a PowerBead Block (Qiagen) containing 0.1 mm glass beads and 450 µL RLT buffer (Qiagen) plus 2-beta mercaptoethanol (Sigma). Blocks were then sealed and homogenized using TissueLyser (Qiagen) at 25 Hz for 2 min. Blocks were then rotated, and an additional homogenization step was performed. Lysate was removed from the block after centrifugation, and RNA/DNA was simultaneously isolated with an All-Prep 96 HT kit (Qiagen) following the manufacturer’s instructions.

#### Bulk RNA sequencing, alignment, and analysis

Please see the supplemental methods for a description on complete bulk RNA processing, alignment, and analysis. Briefly, libraries were generated using the Illumina TruSeq mRNA stranded kit. The libraries were loaded onto NovaSeq 6000 and sequenced with a total read length of 1 × 50 bp, with each sample reaching at least 27 million single-end reads. Once sequencing was complete, the reads were QCed before proceeding to downstream analysis. RNA-seq data was processed using HTSeqGenie (50). The reads were filtered and aligned to Genome Reference Consortium Human Build 38 (GRCh38) using GSNAP (51), and uniquely mapping reads to gene models present in the GENCODE basic annotation set (v. 27) were used to generate the gene count matrix and downstream analyses. Analysis and visualizations were performed in R version 4.2.0 (https://www.r-project.org/). The edgeR package was used to perform trimmed mean of m-values (TMM) normalization and log transform counts. Differential gene expression analysis was performed using the edgeR package with the voom-limma framework, with the subject introduced as a blocking factor. Genes were reported as statistically significant if they had an FDR value below 0.05 after adjusting for multiple comparisons using the Benjamini–Hochberg method.

### Single-cell RNA-seq atlas

#### Patient and tissue sample collection

The subjects were enrolled in the Stanford Inflammatory Bowel Disease (IBD) Registry used for all ulcerative colitis (UC) patients and healthy controls under Institutional Review Board (IRB) Protocol 52317 at Stanford University School of Medicine. Informed consent was obtained from all patients according to the protocol, and the plans for sequencing, data storage, and publication were approved by the Stanford IRB. UC patients had a clinical diagnosis of ulcerative colitis and were observed to have active disease. Biopsies were obtained during endoscopy, using biopsy cold forceps used in standard care. The endoscopist visually evaluated the presence or absence of inflammation during collection using the Mayo Endoscopic Score (MES) to ensure consistency. Four to six fresh biopsies were obtained from inflamed and uninflamed colonic segments from 20 UC patients. Biopsy bites were immediately placed into Advanced DMEM F-12 media (Gibco, catalog number 11320033), placed on wet ice for transport, and immediately processed fresh for single-cell experiments.

#### Single-cell biopsy processing

For details on single-cell biopsy processing, please refer to supplemental methods. Briefly, biopsies were processed using a modified protocol from Smillie et al. (2018) where the epithelial layer and the lamina propria were separated and processed separately (52). The epithelial layer was first dissociated using epithelial dissociation medium and remnant tissue that contained the lamina propria, placed on ice, and dissociated separately using digestion medium.

#### Single-cell RNA sequencing

Epithelial and lamina propria single-cell suspensions were loaded into a BD Rhapsody™ Cartridge (BD Biosciences; Cat. No. 633733) at a 1:1 ratio with a target total number of 40,000–60,000 cells. Following the manufacturer’s instructions, single-cell capture, barcoding, lysis, and cDNA synthesis were performed with the BD Rhapsody™ Express Single-Cell Analysis System. Following the manufacturer’s instructions, whole transcriptome analysis (WTA) libraries were indexed and prepared using the BD Rhapsody™ WTA Amplification Kit (BD Biosciences, cat. no. 633801). The libraries were sequenced on Illumina MiSeq, HiSeq 2500, NextSeq 2000, and NovaSeq 6000 (Illumina) with the following configuration: read 1, 75 bp, i7 index: 8 bp, i5 index: none; read 2, 75 bp.

#### Single-cell RNA analysis

Please see supplemental methods for a complete description of the analysis of single-cell RNA-seq data. Briefly, individual samples were aligned to a human reference genome using the BD Rhapsody Seven Bridges pipeline and then processed using the Seurat package V4 (53). The samples were individually QCed and then integrated and harmonized into a larger object using the Harmony package (54). Doublets were manually removed through a manual iterative process and then annotated using a combination of the SCimilarity package and manual annotation (55). The annotation marker genes are described in supplemental methods (**Supplementary Table S5**). After generating a QCed, integrated, and annotated object, cell type abundances were calculated between uninflamed and inflamed samples at the patient level modeled by a negative binomial distribution with the edgeR package. Cell types were reported as differentially abundant between inflamed and non-inflamed samples if the FDR value was less than 0.05 after adjusting for multiple comparisons with the Benjamini–Hochberg method. Cell-specific gene modules were identified by identifying marker genes that were unique to that specific cluster and not expressed by more than 2% of cells outside that cluster. Trajectory analysis was performed on neutrophil subsets using the Monocole 3 package (56–58) by subsetting neutrophils, reclustering, generating a neutrophil-specific embedding, and calculating the root of the trajectory using the most proximal node to early cell states identified by the get_earliest_principal_node_function. A logistic regression model was developed to identify associations between MCS remission and changes in neutrophil states, adjusting for patient-reported sex. Lastly, ligand–receptor analysis was performed using the Cell Chat package to identify communication networks that were distinct between inflamed and uninflamed tissue (59). Interactions were considered significant if the observed ligand–receptor distribution was significantly different than the null distribution after a permutation test with a *p*-value <0.05.

## Data Availability

The single-cell RNA-seq atlas presented in this study is deposited in the Broad Institute Single Cell Portal under accession number SCP3755. Following consultation with Roche Privacy Counsel and an assessment of the current privacy landscape and the risk of data subject re-identification, we are unable to deposit the transcriptomic data from the HIBISCUS studies in a repository or make them available to external researchers due to privacy risks to our data subjects. Further inquiries can be directed to the corresponding author/s.

## Supplementary material

Supplementary Figure 1 Transcriptomic analysis of etrolizumab, adalimumab, and placebo treatment in colonic biopsies **(A–D)**. Expression of selected integrins before (baseline) and after (induction; week 10) adalimumab treatment. Each point represents an individual patient, and pre-and post-treatment trajectories for each patient are shown (Gray bar). Expression values are in normalized counts per million (CPM). Box plots represent the upper and lower quartiles, the middle line represents the median, notches show 1.58x the interquartile range (IQR) divided by the square root of the number of samples measured, and the whiskers extend to the most extreme point no more than 1.5x outside of the interquartile ranges **(E–H)**. Expression of selected integrins before (baseline) and after (induction; week 10) placebo treatment **(I)**. Comparison of log2 fold changes between placebo (x-axis) and etrolizumab (y-axis). Each point represents a gene, colored blue for genes significantly up-regulated after treatment (fold change > 1.5x at an FDR of 0.05) and colored red for genes significantly down-regulated after treatment (fold change < -1.5x at an FDR of 0.05) **(J)**. Volcano plots showing log2 fold change on the x-axis and -log10 p-value on the y-axis for changes in gene expression after placebo treatment for 10 weeks. Each point represents a gene, colored as in **(I)** above **(K).** Venn diagrams comparing the number of genes significantly down-regulated or up-regulated after treatment with etrolizumab or adalimumab for 10 weeks and the number of genes significantly down-regulated or up-regulated after treatment with etrolizumab or placebo for 10 weeks **(L–O)**. Expression of selected neutrophil-associated or epithelium-associated genes before (baseline) or after (induction; week 10) adalimumab treatment. Each point represents a sample collected from a patient at the respective time point, and patient measurements are joined by a line. Boxes are colored by remission status of the patient at week 10. Boxes represent the upper and lower quartile, the middle line represents the median, and the whiskers extend to the most extreme point no more than 1.5x the interquartile range from the box boundaries. For all panels, stars indicate Benjamini-Hochberg corrected p-values: ns: p > 0.01; *: p < 0.01; ** p < 0.001; *** p < 0.0001 **(P–S)**. Expression of selected neutrophil-associated or epithelium-associated genes before (baseline) or after (induction; week 10) placebo treatment.

Supplementary Figure 2 Neutrophils are elevated in inflamed and more severe patient samples. **(A)** Patient cell type percentage distribution across inflamed and uninflamed samples at the broadest level of annotation. Each bar represents a sample of colonic tissue from a patient, and colors indicate the percent of cell type within stacked barplot n = 19 inflamed and 18 uninflamed. **(B)** Summary statistics for differential abundance analysis at Annotation level 2 are shown. Dotplot depicting log2 fold change (x-axis) and -log10(FDR) (y-axis) for each cell type at annotation level 2 across all patients. Dots in blue are elevated in uninflamed samples, and dots in orange are elevated in inflamed samples. All dots above the dashed line are significantly elevated with an FDR value of < 0.05. **(C)** Uniform manifold approximation plots (UMAPs) showing Patient ID, Biopsy inflammation status, and Mayo Endoscopy subscore across all patients. **(D)** Box plots ordered by FDR, depicting differential abundance analysis at the patient level for all cell types at an intermediate level of granularity, split by inflammation status. The y-axis represents the percentage of each cell type out of the total cells in the sample. Each dot represents a sample from a patient. Blue boxes represent uninflamed samples, and orange boxes represent inflamed samples. FDR and fold change are displayed in the upper left corner of each plot and were calculated separately (see Methods).

Supplementary Figure 3 Single-cell gene signatures derived to deconvolve bulk transcriptomics data. **(A)** Single-cell-derived cell-specific gene modules applied to bulk transcriptomics data are shown. Each point represents the expression of a gene in a cell population. The size of the point indicates the proportion of cells expressing the gene, and the color indicates the centered and scaled expression level.

Supplementary Figure 4 Cellular specificity of selected integrins in UC tissue and correlation between and changes in gene modules in adalimumab and placebo-treated patients. **(A)** Heatmap of Spearman correlation between signature scores for individual samples. **(B)** Effect of etrolizumab on individual cell type expression signatures (x-axis) compared with differential abundance of the cell type in inflamed vs uninflamed biopsies (y-axis). Each point represents an individual cell population. **(C)** Effect of treatment on genes specific for individual cell populations in remitters or non-remitters after adalimumab treatment. Boxes represent the log2 fold change between baseline and week 10 for either non-remitters or remitters. Cell populations are sorted according to the order in **Figure 3B**. **(D)** Each point represents the expression of an integrin gene on a cell population. The size of the point indicates the proportion of cells expressing the gene, and the color indicates the centered and scaled expression level. **(E)** Expression of neutrophil signature score before and after treatment with placebo. Each point represents a patient sample before (baseline) or after (induction, week 10) treatment. Samples collected from the same patient are linked by a line. **(F)** Expression of inflammatory fibroblast signature score before and after treatment with placebo. Each point represents a patient sample before (baseline) or after (induction, week 10) treatment. Samples collected from the same patient are linked by a line. **(G)** Comparison of log2 fold change in neutrophil gene expression signature (y-axis) with change in Mayo Clinic score (x-axis) after treatment with placebo. Each point represents an individual patient, colored by remission status at week 10. **(H)**. Comparison of log2 fold change in inflammatory fibroblast gene expression signature (y-axis) with change in Mayo Clinic score (x-axis) after treatment with placebo. Each point represents an individual patient, colored as in **(D)**.

Supplementary Figure 5 Anti-TNF resistance modules are expressed by inflammatory fibroblast and neutrophil subsets **(A)** Heatmap showing the Z-Score of average expression of TNF resistance module genes M4 and M5 from Friedrich et al. (12) at level 2 annotations.

Supplementary Figure 6 Heterogeneity of neutrophils in UC tissue biopsies. **(A)** UMAP projection of neutrophil subsets, including low RNA neutrophils. Each point represents a cell, colored by the respective subset it was classified as. **(B)** Differential abundance of neutrophils across inflamed and uninflamed tissue. Each point represents a sample. The y-axis shows the fraction of each neutrophil subset relative to the total number of neutrophils in that sample. Fold changes and FDR values were determined as described in the methods. **(C)** Heatmap showing the Z-Score of average expression of genes used to identify neutrophil subsets from Garrido-Trigo et al. (18) mapped onto our neutrophil subsets. Rows are ordered from the earliest subset to the latest subset in pseudotime, and columns are hierarchically clustered using Euclidean distance. **(D)** Single cell-derived gene modules specific to neutrophil subsets are shown across all cell populations. Each point represents the expression of a gene in a cell population. The size of the point indicates the proportion of cells expressing the gene, and the color indicates the centered and scaled expression level. **(E)** ROC curves for the association of baseline levels of neutrophil subsets with remission. **(F)** UMAP projection of neutrophil subsets colored by the inferred pseudotime. The inferred trajectory is shown in black lines. The white circle indicates the starting point for the trajectory inference **(G)** Expression of BPI and HP along the neutrophil pseudotime axis. Each dot represents a cell with black lines indicating the expression of each gene along pseudotime. The UMAPs below show the expression of BPI and MMP9 in neutrophils.

Supplementary Figure 7 Gene expression across pseudotime for neutrophil subsets. **(A)** Heatmap showing the Z-Score of log normalized gene expression values for genes with a q-value of < 0.001 and a Moran’s I value > 0.05 after testing differential gene expression across pseudotime. Genes are ordered across pseudotime. Pseudotime consists of bins, each containing 100 cells.

Supplementary Figure 8 Neutrophil interactions in the UC uninflamed tissue. **(A)** Differential number of inferred interactions between inflamed and uninflamed tissue based on cellChat. Adventitial fibroblast cells in inflamed tissue were excluded from cell-cell interaction analysis due to the low number of cells identified (8 cells). **(B)** Heatmap of signaling pathways CXCL and IL1 showing significant communications between neutrophil subpopulations and fibroblasts and endothelial. We observe fewer communication probabilities in the uninflamed tissue than in the inflamed tissue. The color in the heatmap is proportional to the overall communication probability between each cell type. **(C)** Selected significant ligand-receptors as incoming signaling interactions to neutrophil subpopulations from fibroblasts and endothelial subsets in the uninflamed tissue. **(D)**. Significant ligand-receptors were selected as outgoing signaling interactions between neutrophil subpopulations, fibroblasts, and endothelial subsets in the uninflamed tissue.
