# Supplementary figures and images for: A single-cell atlas of ulcerative colitis reveals neutrophil–stromal circuits linked to biologic therapy resistance
Source: Front Immunol. 2026 Jun 11;17:1705328. doi: 10.3389/fimmu.2026.1705328 (PMC13294476; doi:10.3389/fimmu.2026.1705328)

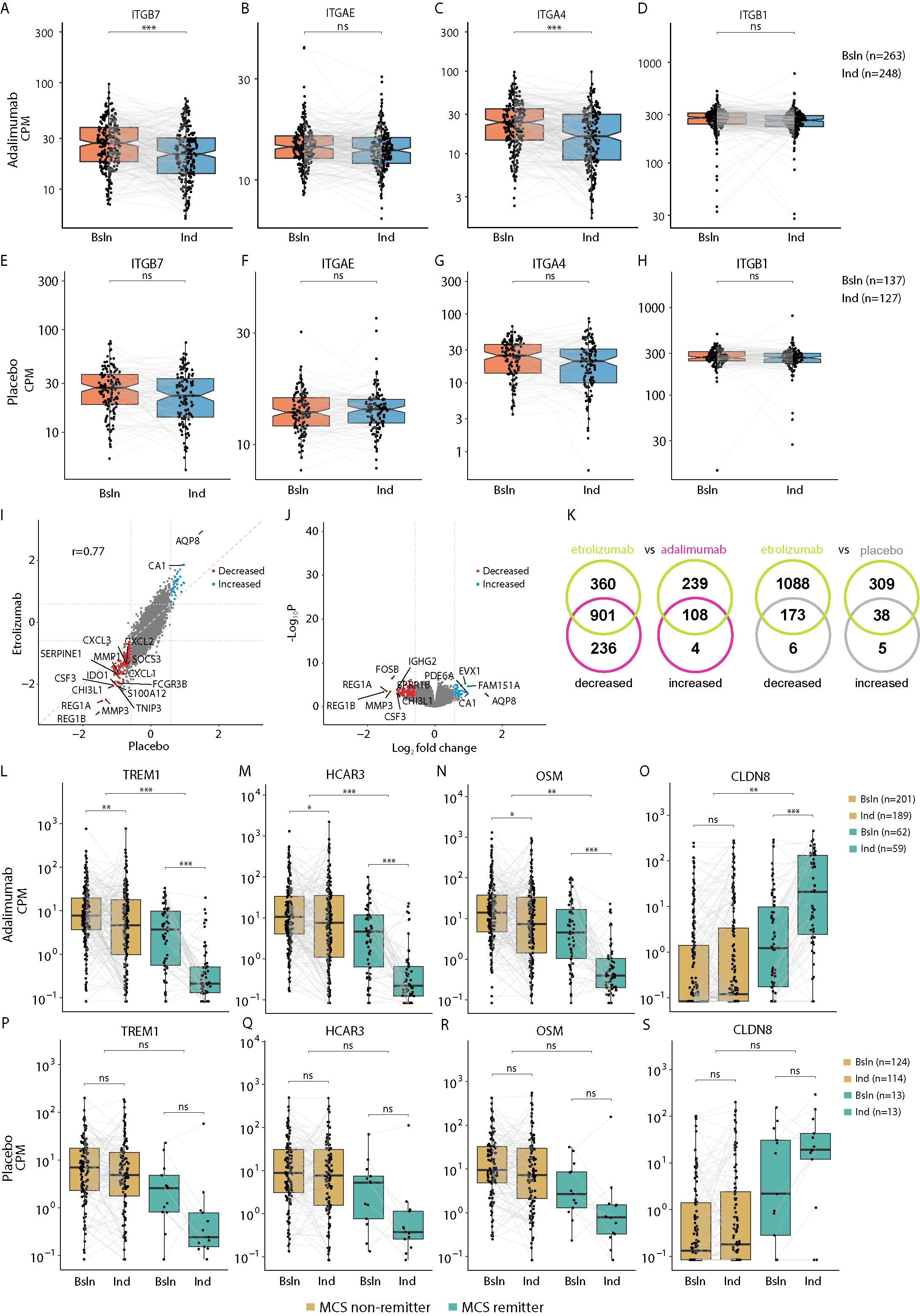

Supplement: Supplementary Figure 1 — Transcriptomic analysis of etrolizumab, adalimumab, and placebo treatment in colonic biopsies (A–D). Expression of selected integrins before (baseline) and after (induction; week 10) adalimumab treatment. Each point represents an individual patient, and pre-and post-treatment trajectories for each patient are shown (Gray bar). Expression values are in normalized counts per million (CPM). Box plots represent the upper and lower quartiles, the middle line represents the median, notches show 1.58x the interquartile range (IQR) divided by the square root of the number of samples measured, and the whiskers extend to the most extreme point no more than 1.5x outside of the interquartile ranges (E–H). Expression of selected integrins before (baseline) and after (induction; week 10) placebo treatment (I). Comparison of log2 fold changes between placebo (x-axis) and etrolizumab (y-axis). Each point represents a gene, colored blue for genes significantly up-regulated after treatment (fold change > 1.5x at an FDR of 0.05) and colored red for genes significantly down-regulated after treatment (fold change < -1.5x at an FDR of 0.05) (J). Volcano plots showing log2 fold change on the x-axis and -log10 p-value on the y-axis for changes in gene expression after placebo treatment for 10 weeks. Each point represents a gene, colored as in (I) above (K). Venn diagrams comparing the number of genes significantly down-regulated or up-regulated after treatment with etrolizumab or adalimumab for 10 weeks and the number of genes significantly down-regulated or up-regulated after treatment with etrolizumab or placebo for 10 weeks (L–O). Expression of selected neutrophil-associated or epithelium-associated genes before (baseline) or after (induction; week 10) adalimumab treatment. Each point represents a sample collected from a patient at the respective time point, and patient measurements are joined by a line. Boxes are colored by remission status of the patient at week 10. Boxes represent th [file Image1.tif]

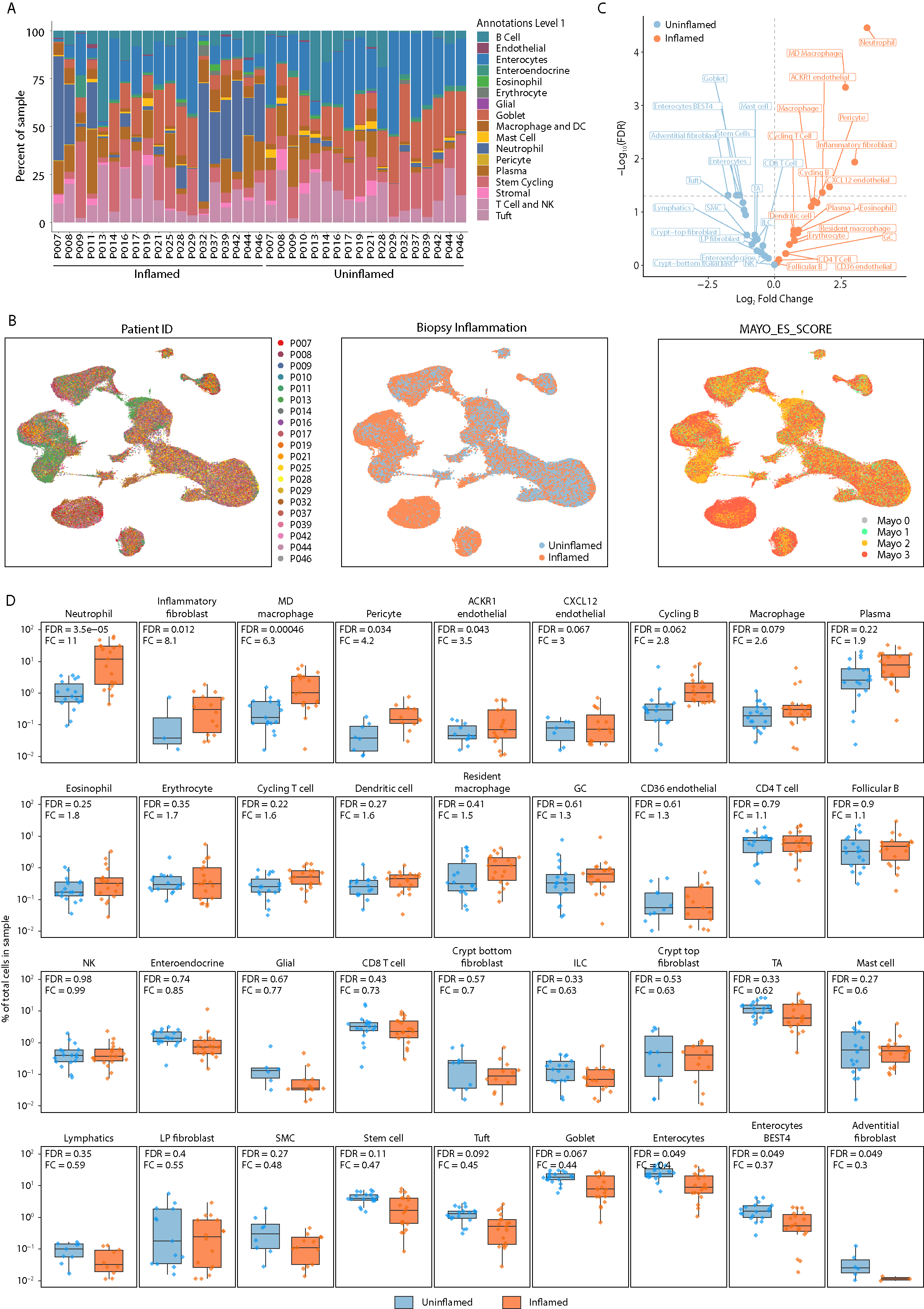

Supplement: Supplementary Figure 2 — Neutrophils are elevated in inflamed and more severe patient samples. (A) Patient cell type percentage distribution across inflamed and uninflamed samples at the broadest level of annotation. Each bar represents a sample of colonic tissue from a patient, and colors indicate the percent of cell type within stacked barplot n = 19 inflamed and 18 uninflamed. (B) Summary statistics for differential abundance analysis at Annotation level 2 are shown. Dotplot depicting log2 fold change (x-axis) and -log10(FDR) (y-axis) for each cell type at annotation level 2 across all patients. Dots in blue are elevated in uninflamed samples, and dots in orange are elevated in inflamed samples. All dots above the dashed line are significantly elevated with an FDR value of < 0.05. (C) Uniform manifold approximation plots (UMAPs) showing Patient ID, Biopsy inflammation status, and Mayo Endoscopy subscore across all patients. (D) Box plots ordered by FDR, depicting differential abundance analysis at the patient level for all cell types at an intermediate level of granularity, split by inflammation status. The y-axis represents the percentage of each cell type out of the total cells in the sample. Each dot represents a sample from a patient. Blue boxes represent uninflamed samples, and orange boxes represent inflamed samples. FDR and fold change are displayed in the upper left corner of each plot and were calculated separately (see Methods). [file Image2.tif]

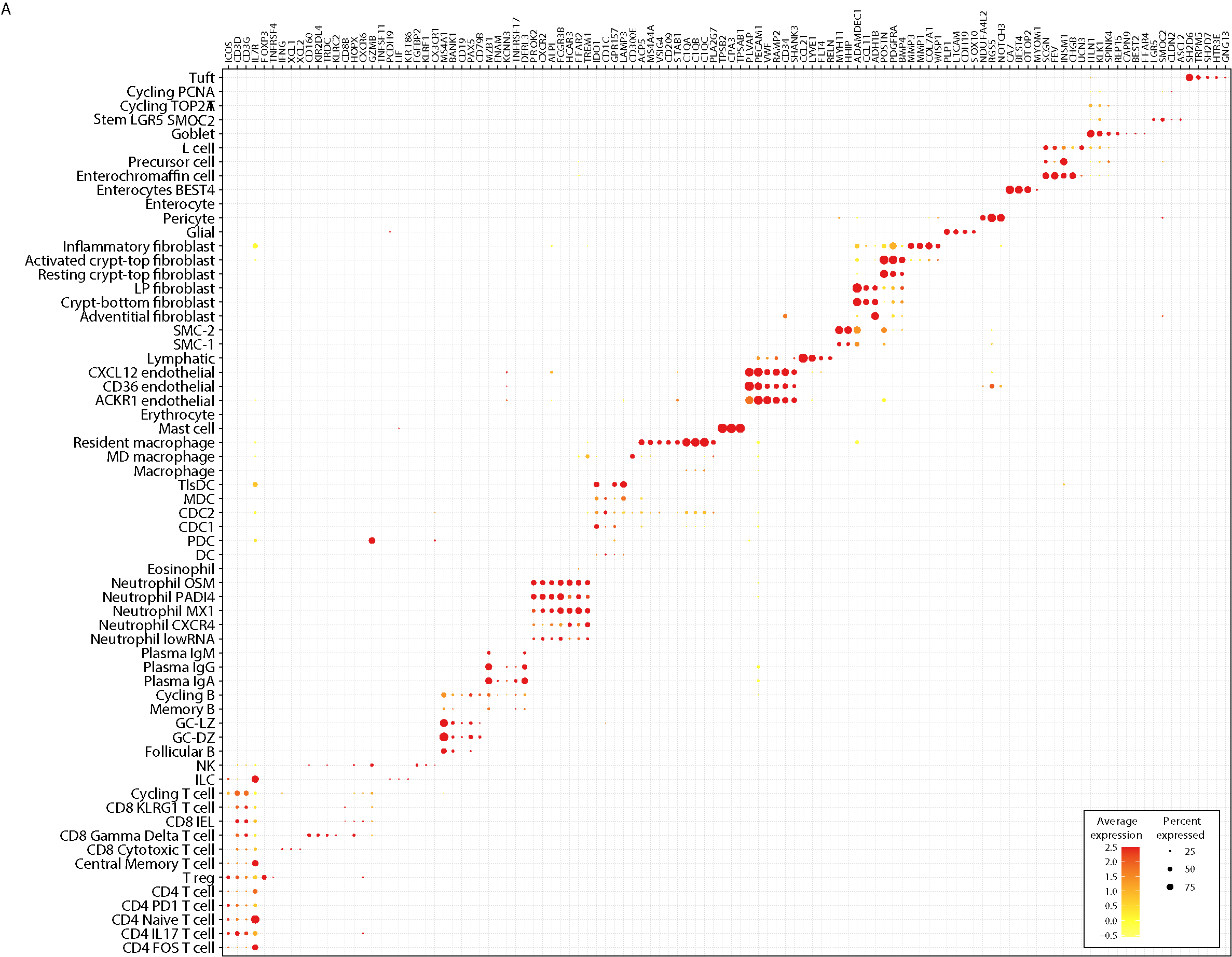

Supplement: Supplementary Figure 3 — Single-cell gene signatures derived to deconvolve bulk transcriptomics data. (A) Single-cell-derived cell-specific gene modules applied to bulk transcriptomics data are shown. Each point represents the expression of a gene in a cell population. The size of the point indicates the proportion of cells expressing the gene, and the color indicates the centered and scaled expression level. [file Image3.tif]

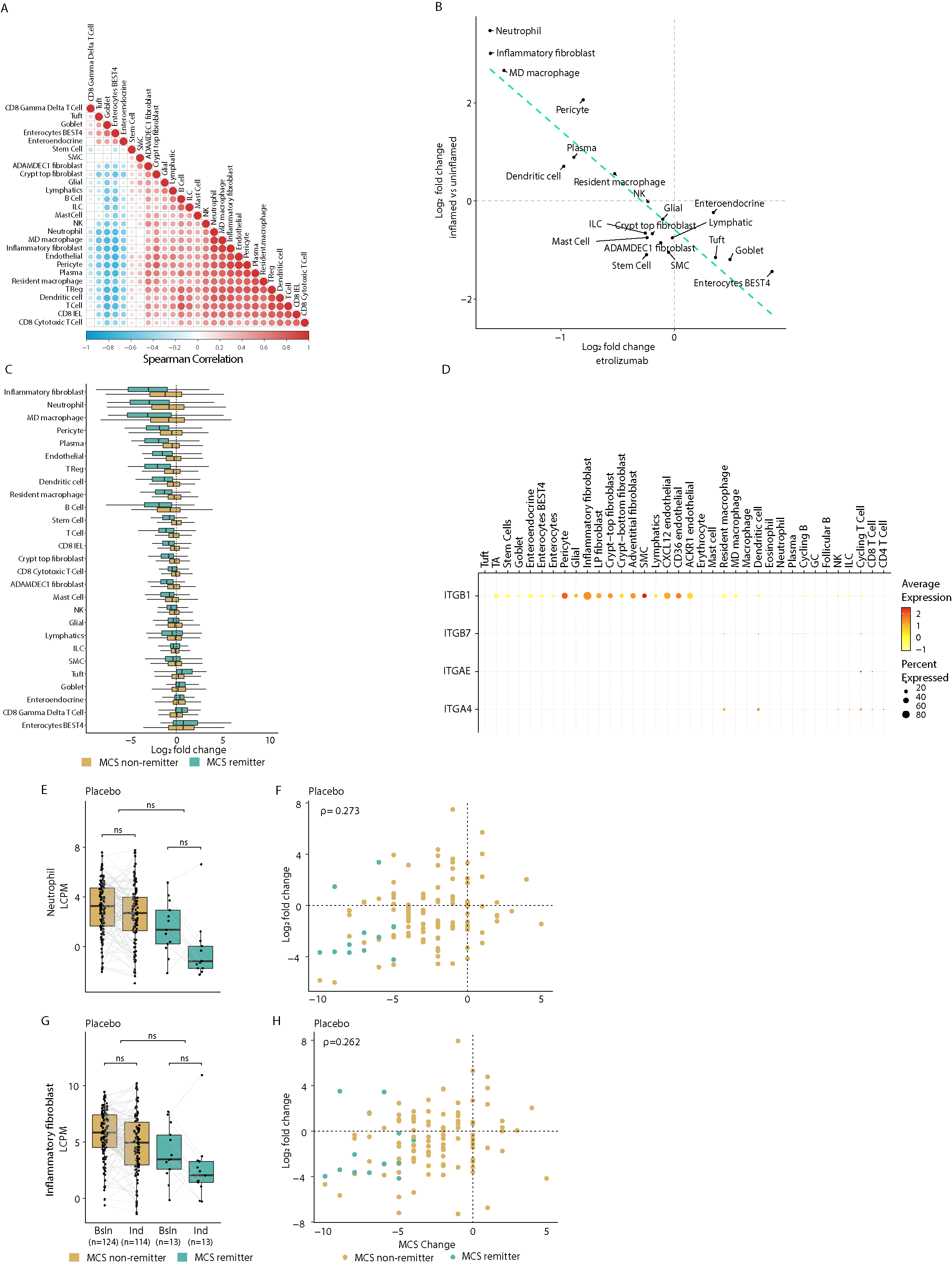

Supplement: Supplementary Figure 4 — Cellular specificity of selected integrins in UC tissue and correlation between and changes in gene modules in adalimumab and placebo-treated patients. (A) Heatmap of Spearman correlation between signature scores for individual samples. (B) Effect of etrolizumab on individual cell type expression signatures (x-axis) compared with differential abundance of the cell type in inflamed vs uninflamed biopsies (y-axis). Each point represents an individual cell population. (C) Effect of treatment on genes specific for individual cell populations in remitters or non-remitters after adalimumab treatment. Boxes represent the log2 fold change between baseline and week 10 for either non-remitters or remitters. Cell populations are sorted according to the order in Figure 3B. (D) Each point represents the expression of an integrin gene on a cell population. The size of the point indicates the proportion of cells expressing the gene, and the color indicates the centered and scaled expression level. (E) Expression of neutrophil signature score before and after treatment with placebo. Each point represents a patient sample before (baseline) or after (induction, week 10) treatment. Samples collected from the same patient are linked by a line. (F) Expression of inflammatory fibroblast signature score before and after treatment with placebo. Each point represents a patient sample before (baseline) or after (induction, week 10) treatment. Samples collected from the same patient are linked by a line. (G) Comparison of log2 fold change in neutrophil gene expression signature (y-axis) with change in Mayo Clinic score (x-axis) after treatment with placebo. Each point represents an individual patient, colored by remission status at week 10. (H). Comparison of log2 fold change in inflammatory fibroblast gene expression signature (y-axis) with change in Mayo Clinic score (x-axis) after treatment with placebo. Each point represents an individual patient, colored as in (D). [file Image4.tif]

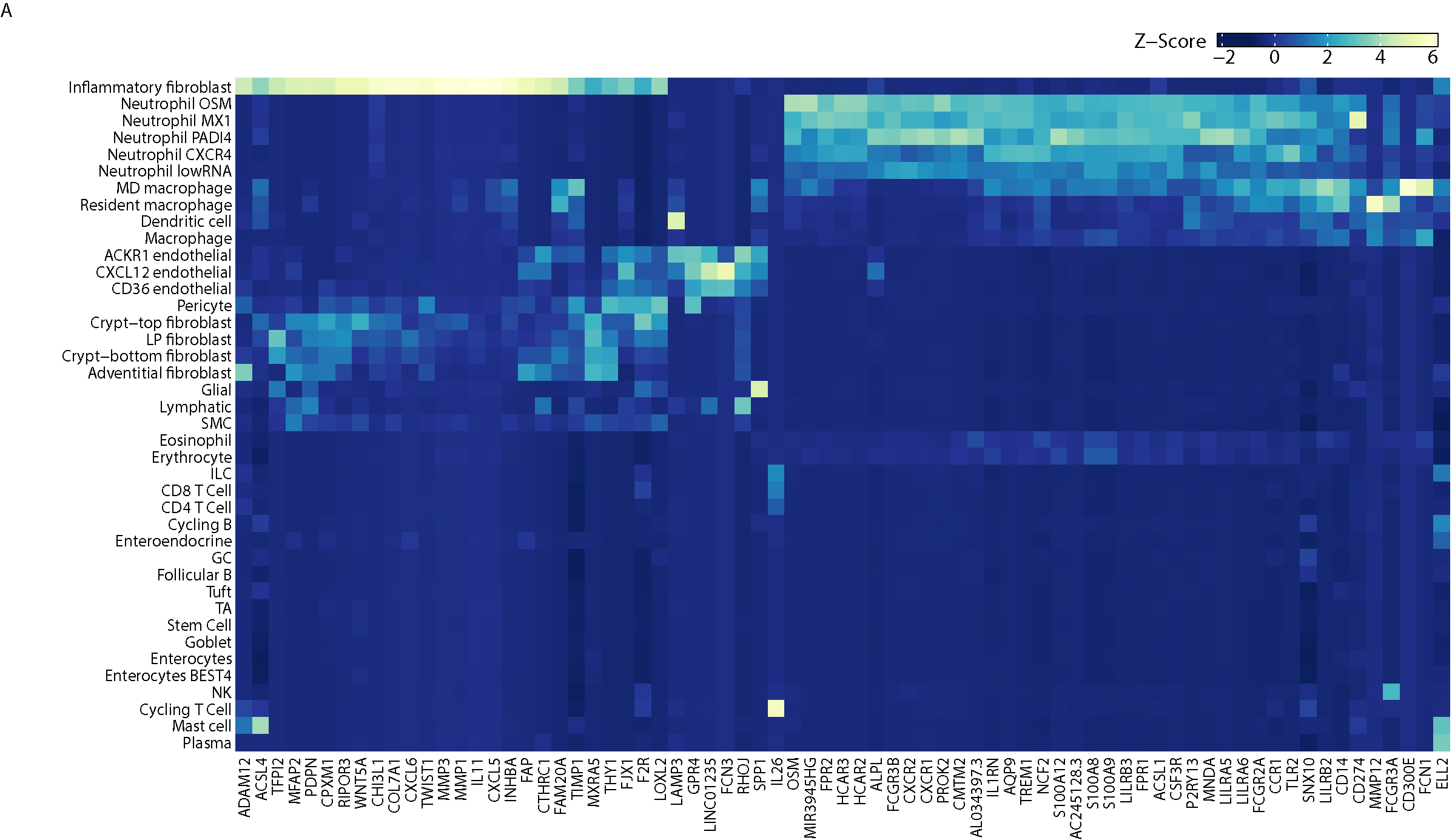

Supplement: Supplementary Figure 5 — Anti-TNF resistance modules are expressed by inflammatory fibroblast and neutrophil subsets (A) Heatmap showing the Z-Score of average expression of TNF resistance module genes M4 and M5 from Friedrich et al. (12) at level 2 annotations. [file Image5.tif]

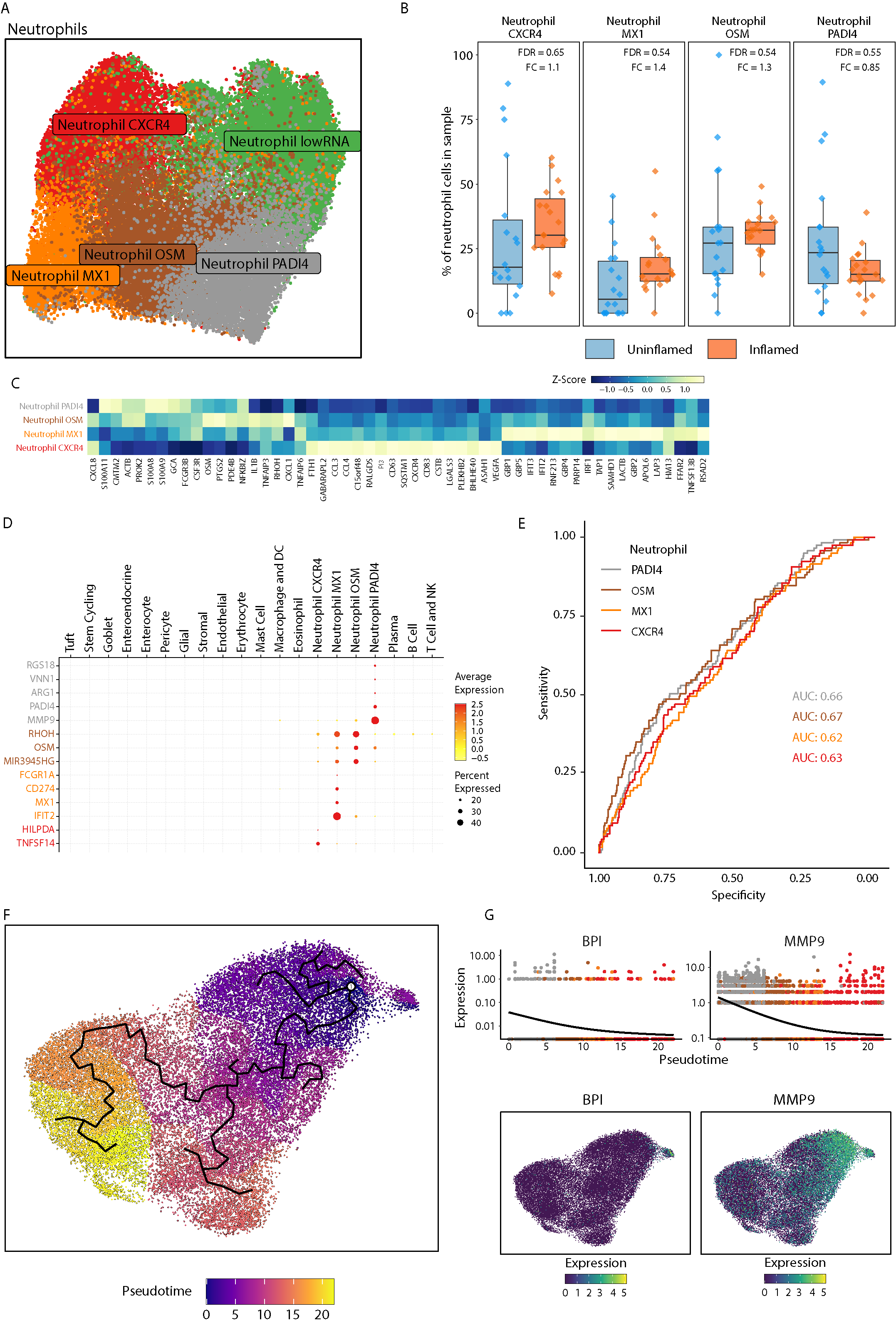

Supplement: Supplementary Figure 6 — Heterogeneity of neutrophils in UC tissue biopsies. (A) UMAP projection of neutrophil subsets, including low RNA neutrophils. Each point represents a cell, colored by the respective subset it was classified as. (B) Differential abundance of neutrophils across inflamed and uninflamed tissue. Each point represents a sample. The y-axis shows the fraction of each neutrophil subset relative to the total number of neutrophils in that sample. Fold changes and FDR values were determined as described in the methods. (C) Heatmap showing the Z-Score of average expression of genes used to identify neutrophil subsets from Garrido-Trigo et al. (18) mapped onto our neutrophil subsets. Rows are ordered from the earliest subset to the latest subset in pseudotime, and columns are hierarchically clustered using Euclidean distance. (D) Single cell-derived gene modules specific to neutrophil subsets are shown across all cell populations. Each point represents the expression of a gene in a cell population. The size of the point indicates the proportion of cells expressing the gene, and the color indicates the centered and scaled expression level. (E) ROC curves for the association of baseline levels of neutrophil subsets with remission. (F) UMAP projection of neutrophil subsets colored by the inferred pseudotime. The inferred trajectory is shown in black lines. The white circle indicates the starting point for the trajectory inference (G) Expression of BPI and HP along the neutrophil pseudotime axis. Each dot represents a cell with black lines indicating the expression of each gene along pseudotime. The UMAPs below show the expression of BPI and MMP9 in neutrophils. [file Image6.tif]

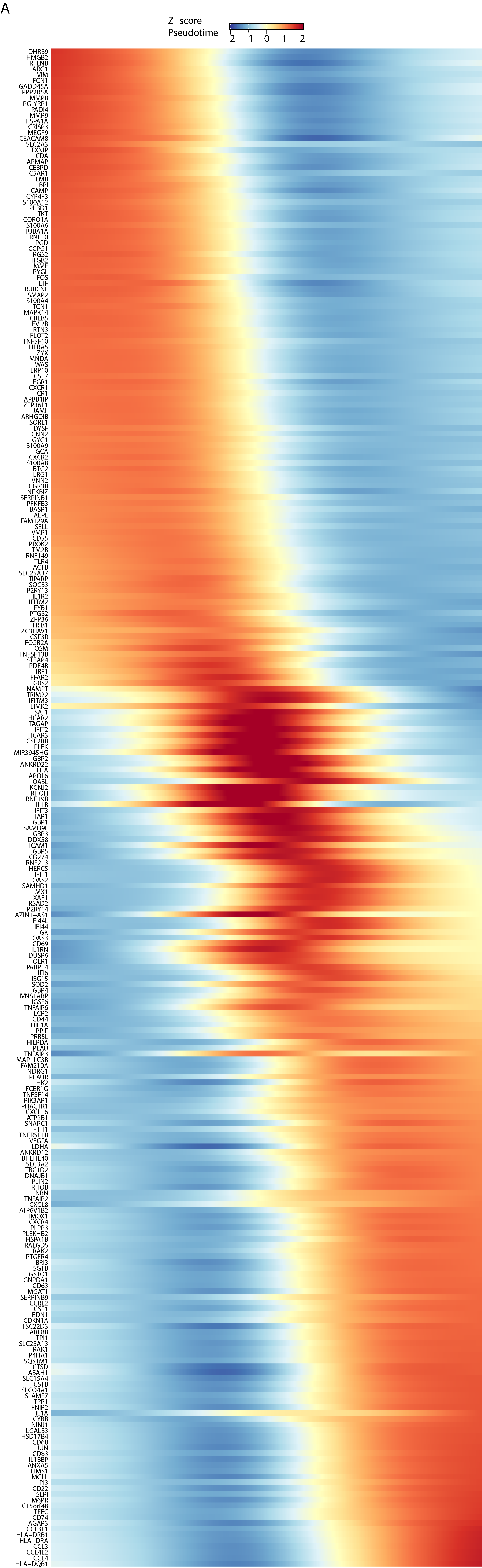

Supplement: Supplementary Figure 7 — Gene expression across pseudotime for neutrophil subsets. (A) Heatmap showing the Z-Score of log normalized gene expression values for genes with a q-value of < 0.001 and a Moran’s I value > 0.05 after testing differential gene expression across pseudotime. Genes are ordered across pseudotime. Pseudotime consists of bins, each containing 100 cells. [file Image7.tif]

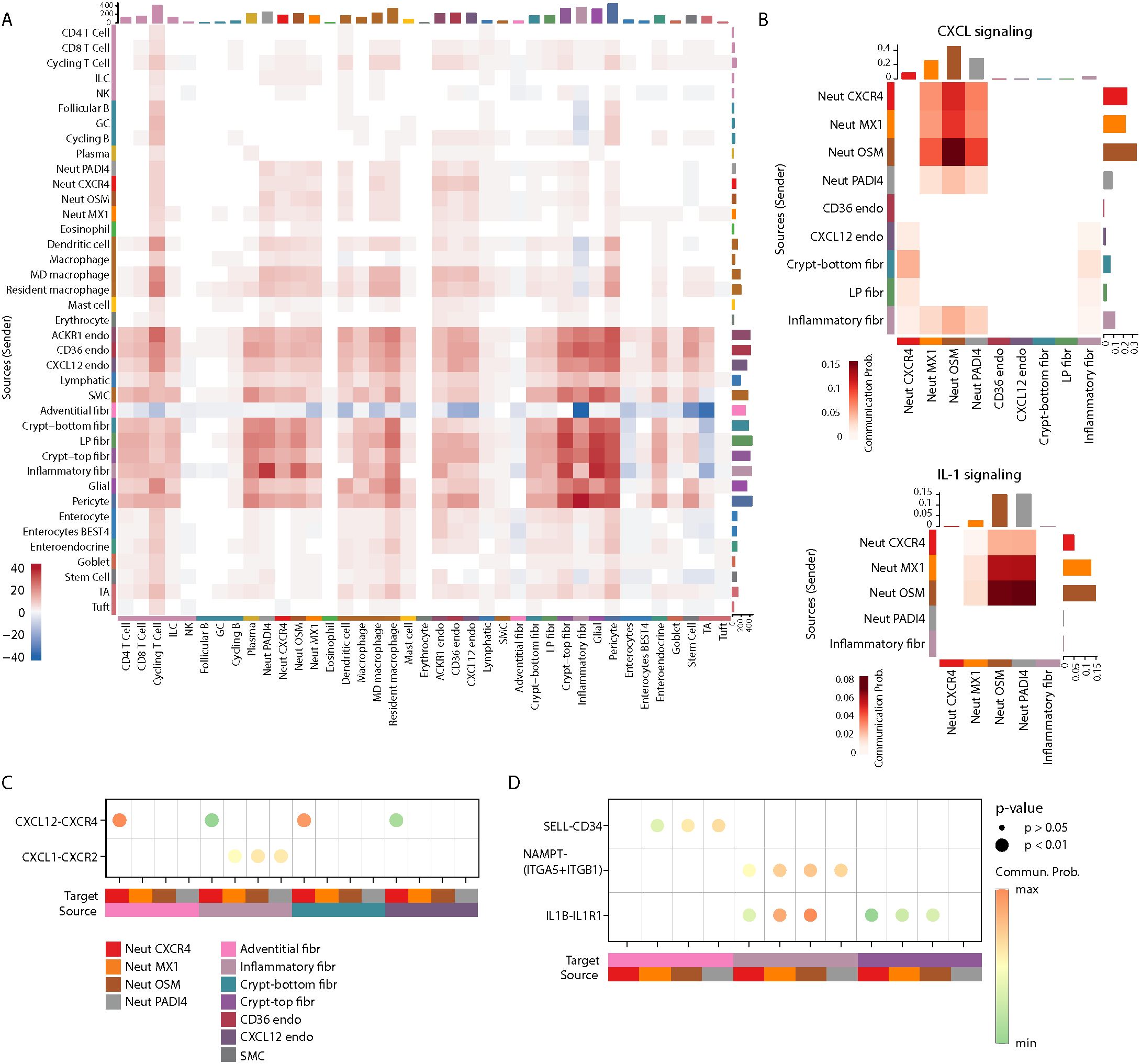

Supplement: Supplementary Figure 8 — Neutrophil interactions in the UC uninflamed tissue. (A) Differential number of inferred interactions between inflamed and uninflamed tissue based on cellChat. Adventitial fibroblast cells in inflamed tissue were excluded from cell-cell interaction analysis due to the low number of cells identified (8 cells). (B) Heatmap of signaling pathways CXCL and IL1 showing significant communications between neutrophil subpopulations and fibroblasts and endothelial. We observe fewer communication probabilities in the uninflamed tissue than in the inflamed tissue. The color in the heatmap is proportional to the overall communication probability between each cell type. (C) Selected significant ligand-receptors as incoming signaling interactions to neutrophil subpopulations from fibroblasts and endothelial subsets in the uninflamed tissue. (D). Significant ligand-receptors were selected as outgoing signaling interactions between neutrophil subpopulations, fibroblasts, and endothelial subsets in the uninflamed tissue. [file Image8.tif]
