# Supplementary material for: A single-cell atlas of ulcerative colitis reveals neutrophil–stromal circuits linked to biologic therapy resistance
Source: Front Immunol. 2026 Jun 11;17:1705328. doi: 10.3389/fimmu.2026.1705328 (PMC13294476; doi:10.3389/fimmu.2026.1705328)
Supplement: Supplementary file 9 [file Supplementaryfile1.docx]

### **SUPPLEMENTA**RY **METHODS**

#### **Clinical trial bulk RNA-sequencing**

##### *Bulk RNA sequencing*

Purified RNA isolated from colonic pinch biopsies was assessed for quantity and purity using Qubit and integrity with Agilent Tapestation. RNA of high quality then proceeded to library preparation with the Illumina TruSeq mRNA stranded kit. The resulting libraries were then evaluated for quantity (Quant-It) and fragment size determination (Agilent Tapestation). Successfully generated libraries were loaded onto a NovaSeq 6000 instrument and sequenced with a total read length of 1x50bp, each sample reaching at least 27 million single-end reads. Once sequencing was complete, the quality of the reads was assessed for clusters passing the filter, Q30 scores, error rate, cluster density, and read distribution before proceeding further for downstream workflow analysis.

##### Bulk RNA-seq alignment and differential expression analysis

RNA-seq data was processed using HTSeqGenie [*(1)*](https://www.zotero.org/google-docs/?n6t50A). Reads were filtered and aligned to Genome Reference Consortium Human Build 38 (GRCh38) using GSNAP [*(2)*](https://www.zotero.org/google-docs/?BQ3Stg), and uniquely mapping reads to gene models present in the GENCODE basic annotation set (v. 27) were used for generating the gene count matrix and downstream analyses. Additional analysis and visualizations were performed in R version 4.2.0 (<https://www.r-project.org/>). The EdgR package was used for preprocessing, including filtering of low-count genes, trimmed mean of m-values (TMM) normalization, and log counts per million (lcpm) transformation of counts. Differential gene expression analysis was performed using the Limma-Voom framework with the subject introduced as a blocking factor.

#### Single-cell RNA-seq atlas

##### *Epithelial layer dissociation*

On arrival, biopsy bites were washed twice in cold PBS. Tissue was then added to 5 mL of an EDTA-enriched epithelial dissociation medium [HBSS without Ca^2+^Mg^2+^ (Life Technologies #88284), HEPES 10 mM (Life Technologies #15630106), EDTA 10 mM (Life Technologies #AM9260G], 100 U/ml penicillin (ThermoFisher #15140-122), 100 mg/mL streptomycin (ThermoFisher, #15140-122), FBS 2% + freshly supplemented with 100 µL of 0.5M EDTA] and placed in a rotisserie incubator at 37°C for 15 minutes. Following incubation, the tissue rested on ice for 10 minutes and was shaken vigorously for 15 seconds. Remnant tissue was removed and placed on ice-cold PBS for downstream dissociation of the lamina propria layer. The remaining supernatant containing the epithelial layer was spun at 800 g for 5 minutes, resuspended in 1 mL of epithelial dissociation medium, and transferred to a 1.5 mL Eppendorf tube. The epithelial solution was spun down at 1000 g for 2 minutes and then resuspended in 1 mL of TrypLE express enzyme (ThermoFisher #12604013) for 5 minutes at 37°C, followed by gentle trituration with a P1000 pipette. Epithelial cells were spun down at 1000 g for 2 minutes, resuspended in 200 µL of epithelial cell solution, and placed on ice for 3 minutes before triturating with a P1000 pipette. The solution was filtered into a new Eppendorf tube through a 100 µM cell strainer (Falcon/VWR #21008-949). The epithelial cells were then spun down at 1000 g for 5 minutes, resuspended in 1 mL of PBS with 0.04% BSA, and kept on ice until single-cell experiments.

##### *Lamina propria layer dissociation*

Tissue saved on ice from epithelial layer digestion was transferred into 5 mL of digestion medium [RPMI 1640, 100 U/ml penicillin (ThermoFisher #15140-122), 100 mg/mL streptomycin (ThermoFisher #15140-122), 50 mg/mL gentamicin (ThermoFisher #15750-060)], FBS 2%, HEPES 10 mM (Life Technologies #15630106), and freshly supplemented with 100 µg/mL of Liberase TM (Roche #5401127001) and DNase I 100 µg/mL (Sigma-Aldrich # LS002060) and placed in a rotisserie incubator at 37°C for 30 minutes. After incubation, the enzymatic dissociation was quenched by adding 1 mL of 100% FBS + and 80 µL of 0.5M EDTA and placed on ice for five minutes. The solution was filtered through a 100 µM filter into a new 50 mL conical tube and then transferred to a new 15 mL Falcon tube. The solution was spun down at 1000 g for 5 minutes, resuspended in 1 mL of PBS with 0.04% BSA, and then transferred to a new 1.5 mL Eppendorf tube. The cells were then spun down at 1000 g for 5 min and resuspended in 1 mL of 1X PBS with 0.04% BSA (Invitrogen #AM2616).

##### *Single Cell RNA Individual library preprocessing*

Samples were aligned to a human reference genome using the Seven Bridges pipeline to generate raw gene cell count matrices for each sample then processed using the Seurat package V4 [*(3)*](https://www.zotero.org/google-docs/?gp3fGy). Low-quality cells were removed from the analysis by excluding cells if they had less than one hundred genes or more than twenty-thousand RNA counts per cell. The standard Seurat pipeline was followed by performing log normalization of the counts and scaling and performing linear dimension reduction analysis using the top two thousand variable features for Principal Component Analysis (PCA). K nearest neighbors were then calculated and communities were detected using a Louvain Jaccard clustering algorithm that used euclidean distance to identify communities from top 30 PCAs to create a Uniform Manifold Approximation and Projection (UMAP) reduction. Libraries were only included if they had an appropriate representation of expected cell types in the gut. For example, it was confirmed that libraries had both immune and epithelial cells before being included for analysis. One inflamed and one uninflamed sample did not pass quality control and was omitted, resulting in 38 overall samples, with 18 pairs of inflamed and uninflamed samples across 20 patients.

##### *Merging and integration*

All sequenced libraries for paired inflamed and uninflamed biopsies were individually processed and sequenced, which can introduce batch-specific effects across different samples. To reduce technical and biological batch variability, samples were first merged and then integrated using the Harmony package [*(4)*](https://www.zotero.org/google-docs/?GaqpUQ). Harmony provides a robust approach that projects cells into a shared embedding space based on cell type. Specifically, the top 50 dimensions were used from PCA reduction analysis to integrate merged biological samples.

##### *Annotation at the single-cell level using SCimilarity*

To annotate communities, the integrated dataset was run through SCimilarity [*(5)*](https://www.zotero.org/google-docs/?diZMoq). SCimilarity leverages existing publicly available scRNA-seq atlases to train a deep-learning model to identify single cells. This model can then be applied to novel instances of single cell data where it calculates the distance between each individual cell and cells within the embedding of the model. Specifically, the “ca.annotate_dataset” function was used to annotate cells at the individual level which calculates the cosine distance between each cell and the Scimilarity embedding using the “2023_01_rep0” model. After annotating cells at the individual level, cell annotations were grouped and assigned to one of the following lineages: T cells, B cells, epithelial cells, stromal cells, and myeloid cells. Each lineage was separated into an individual Seurat object for downstream analysis and manual annotation.

##### Doublet and low-quality cell removal

Doublets were iteratively removed to improve community detection as each lineage was annotated. Separate objects were created based on their cell lineage and doublets were removed, the object was reharmonized, and reclustered. Notably, it was essential to reharmonize each time as it improved gene detection within each cluster. Doublets were classified as cells that coexpressed canonical cell type markers across different lineages. For example, clusters were removed that coexpressed EPCAM, a canonical epithelial cell marker, and CD3E, a canonical T cell marker, from either the T cell or epithelial cell object. This process was repeated until doublet clusters were not detected within each cell lineage.

Since epithelial cells often undergo apoptosis during library preparation, this lineage tended to have much higher levels of mitochondrial reads than other lineages. Therefore, to remove low-quality/apoptotic cells from the epithelial lineage, cells were excluded that contained more than seventy percent of their reads from mitochondria. This drastically reduced the number of doublets and low-quality cells present within the lineage and allowed for more accurate community detection.

##### *Annotation of cell lineages*

After removing doublets and low-quality cells from each lineage, lineage Seurat objects were harmonized and clustered at varying degrees of granularity. Lineage objects were annotated at three different levels of granularity, with level 1 being the least granular and level 3 being the most granular. Cells were manually annotated using existing IBD atlases as references [*(6–8)*](https://www.zotero.org/google-docs/?vlWrBW). Clusters were assigned identities according to the expression of marker genes listed in the cell type marker table Table S9).

Stromal cells were annotated as endothelial cells (PECAM1), fibroblasts (ADAMDEC1, ABCA8, LUM, DCN, POSTN), smooth muscle cells (MYH11, NPNT, HHIP), glial cells (ALDH1A1, S100B) and pericytes (RGS5, NDUFA4L2). Endothelial cells were further clustered and subdivided into ACKR1 endothelial cells (ACKR1), CD36 endothelial cells (CD36), CXCL12 endothelial cells (CXCL12), and lymphatics (LYVE1, CCL21). Fibroblasts were also clustered again and subdivided to adventitial fibroblasts (CD34, PI16, SFRP2), crypt-bottom fibroblasts (WNT2B, RSPO3), lamina propria (LP) fibroblasts (ADAMDEC1, ABCA8, CCL2), crypt-top fibroblasts (PDGFRA, F3, WNT5A, WNT5B) and inflammatory fibroblasts (IL11, IL13RA2, CHI3L1). Additionally, we observed a subset of crypt-top fibroblasts in activated states (PDPN).

Epithelial cells (EPCAM) were annotated as Enterocytes (CA1, CA2, SLC26A2/3, AQP8), Stem cycling (PCNA, LGR5, SMOC2, OLFM4), Goblets (MUC2, TFF1), Enteroendocrine (CHGA), and Tuft cells (SH2D6). BEST4+ Enterocytes (BEST4) were then annotated as a subset of enterocytes. Stem cycling cells were clustered again and divided into Stem cells (PCNA, LGR5) and Transit amplifying (TOP2A).

Myeloid cells were annotated as granulocytes (FCGR3B), mast cells (TPSAB1), red blood cells (HBB), or macrophages/DCs (HLA-DRA). Granulocytes were divided into neutrophils (FCGR3B) and eosinophils (CLC, HLA-DRA). Macrophages/DCs were further divided into resident macrophages (C1QA, C1QB), MD macrophages (VCAN, FCN1), or dendritic cells (CD1C). Dendritic cells were further classified as plasmacytoid DCs (IRF7, TNFRSF21), tertiary lymphoid structure-associated DCs (CCR7, CCL19), conventional DC1 (SLAMF8, CLEC9A), conventional DC2 (CD14, CLEC10A), or myeloid DCs (CD1A, ITGAE). While performing annotation, if a cluster of cells expressed multiple lineage-specific markers at high levels, those cells were marked as doublets and excluded from further analysis.

Assigned cell identities were then independently validated by at least one additional researcher to ensure proper annotation. After annotating cell lineage objects, all cell lineage objects were merged together and harmonized to create one final object with 73 unique communities/cell states at the most granular level.

##### *Annotation of Neutrophil subsets*

To further annotate neutrophils, neutrophils were subset, harmonized, and clustered. Each cluster was annotated using marker gene identification. Similar clusters were assigned the same identity. Neutrophil subsets were determined by looking at the top 10 marker genes for each subgroup and identifying known neutrophil-associated genes.

##### *Differential Abundance Analysis*

Differential abundance analysis was performed to compare the number of cells identified between uninflamed and inflamed conditions in annotated cell subsets. Differential abundance calculates the total number of cells per condition at the per-sample level and tests the null hypothesis that the mean abundance between uninflamed and inflamed samples are equal. This hypothesis was tested using edgeR’s gene-wise negative binomial generalized linear model with a quasi-likelihood test [*(9)*](https://www.zotero.org/google-docs/?NHbh22). Briefly, the Seurat object was converted to a single-cell experiment object and then abundances of each cell type were calculated using base R’s table function. Cells were not filtered out if they had low abundance counts since this would lose specific cell subsets with lower numbers of cells. A model matrix was created with a design using uninflamed vs. inflamed as the covariate in addition to patient samples. Weighted likelihood empirical bayes were used to estimate common, trended, and tagwise negative binomial dispersions using the estimateDisp function. Next, raw library sizes were scaled using edgeR’s calcNormFactors to account for differences in library sample sizes. The data was then fit to a generalized linear model with a negative binomial distribution using the edgeRs glmQLFit function. Lastly, a negative binomial test was performed using edgeRs glmQLFTest function on the fitted data and considered cell types as differentially abundant if they had a false discovery rate value of less than 0.05.

##### *Derivation and application of cell-specific gene modules*

Cell-specific gene modules were derived from the scRNA-seq data by first identifying marker genes for each cell population using the Wilcoxauc function from the presto package [*(10)*](https://www.zotero.org/google-docs/?iqyC19). For each cell population, an initial gene module was created by combining the top 20 marker genes by logFC on cells of interest with the top 20 marker genes by the percentage of cells in the population of interest expressing the gene filtered for markers expressed by at most 2 percent of the other cells. This initial list was further manually refined by removing any non-specific marker genes.

Cell-specific signature scores for each sample were calculated by taking the average of logCPM for the corresponding gene module. Analysis of the association of longitudinal changes with MCS remission or treatment was performed using the voom-limma framework, with the subject introduced as a blocking factor and adjusted for patient-reported sex.

##### *ROC analysis*

A logistic regression model was developed to evaluate the association between MCS remission at induction with the longitudinal log2 fold change for each neutrophil state, adjusted for patient reported sex. Similarly, another logistic regression model was built to assess the association between MCS remission at induction with the baseline levels of each neutrophil state, adjusted for patient-reported sex. The ROC curves were generated by comparing the observed and the predicted MCS remission status based on each regression model.

##### *Trajectory analysis*

Trajectory analysis was performed using Monocle 3 to identify differences in neutrophil subset states [*(11–13)*](https://www.zotero.org/google-docs/?6Jhyhu). First, a Seurat object was created that contained only neutrophils and excluded low RNA neutrophils, which was reharmonized and reclustered to generate new UMAP dimension reduction values. Then the Seurat object was converted to the native Monocle 3 format and size factors were estimated. Next, cells were clustered using the Leiden clustering algorithm with a *k* value 30 using Monocle3’s cluster_cells function. Additionally, principal graphs were identified from the reduced dimensional space using reversed graph embedding with the learn_graph function. To unbiasedly identify root nodes for the origin of the pseudotime trajectories Monocle3’s get_earlierst_principal_node function was used to order cells. This assigned a pseudotime trajectory to all cells within the neutrophil lineage, which was then used for downstream analysis.

To visualize the expression of genes across pseudotime, the dataset was subset to contain only the genes of interest such as CXCR2 and CXCR4. Plots were generated to show the expression of genes as a function of pseudotime using the plot_genes_in_pseudotime function from Monocole3, filtering out for a minimum expression value of 0.5 for each cell to remove lowly expressing cells. The default formula was used which uses a natural cubic spline with 3 degrees of freedom.

To generate a heatmap that shows gene expression across pseudotime for multiple genes, genes were tested for differential expression on the principal graph generated using a UMAP with the graph_test function. This function tests for differential expression based on a low dimensional embedding and principal graph of the scRNA-seq data by calculating p-values, q-values, and Moran’s I values for each gene. To loosely filter for genes that varied significantly over pseudotime a threshold was set at q values < 0.001 and for Moran’s I value > 0.05. A spline was fit with 3 degrees of freedom using the smooth.spline function from the stats package to the log2 count matrix of genes passing threshold. This matrix was then converted to Z-score values and bins were generated for cells across pseudotime containing 100 cells per bin. The binned matrix was used to create a pseudotime expression heatmap. To summarize genes that varied across pseudotime, both the maximum expression of each gene and the bin in which maximum expression was observed were reported.

##### *Cell-cell interactions*

Cell-cell interaction analysis was performed using CellChat [*(14)*](https://www.zotero.org/google-docs/?eja6sL). A Seurat object with all cell types except neutrophil-low RNA was created. We then created cellChat objects for inflamed and uninflamed tissue and a merged object of inflamed vs. uninflamed tissue for differential analysis. IL1B: IL1R1 interaction was not within the cellChatDB database and was manually added based on evidence from [*(15)*](https://www.zotero.org/google-docs/?0PsiPs). The minimum number of cells to create the communication network for a cell population was set to 10. We did not create interactions of Adventitial fibroblast in the inflamed tissue due to low cell numbers (n=8). Analyses were run using default parameters.

### REFERENCES

[1. G. Pau, J. Reeder, HTSeqGenie: A NGS analysis pipeline (2023).](https://www.zotero.org/google-docs/?9eqRuI)

[2. T. D. Wu, S. Nacu, Fast and SNP-tolerant detection of complex variants and splicing in short reads. *Bioinformatics* **26**, 873–881 (2010).](https://www.zotero.org/google-docs/?9eqRuI)

[3. Y. Hao, S. Hao, E. Andersen-Nissen, W. M. Mauck, S. Zheng, A. Butler, M. J. Lee, A. J. Wilk, C. Darby, M. Zager, P. Hoffman, M. Stoeckius, E. Papalexi, E. P. Mimitou, J. Jain, A. Srivastava, T. Stuart, L. M. Fleming, B. Yeung, A. J. Rogers, J. M. McElrath, C. A. Blish, R. Gottardo, P. Smibert, R. Satija, Integrated analysis of multimodal single-cell data. *Cell* **184**, 3573-3587.e29 (2021).](https://www.zotero.org/google-docs/?9eqRuI)

[4. I. Korsunsky, N. Millard, J. Fan, K. Slowikowski, F. Zhang, K. Wei, Y. Baglaenko, M. Brenner, P. Loh, S. Raychaudhuri, Fast, sensitive and accurate integration of single-cell data with Harmony. *Nat. Methods* **16**, 1289–1296 (2019).](https://www.zotero.org/google-docs/?9eqRuI)

[5. G. Heimberg, T. Kuo, D. DePianto, T. Heigl, N. Diamant, O. Salem, G. Scalia, T. Biancalani, S. Turley, J. Rock, H. C. Bravo, J. Kaminker, J. A. V. Heiden, A. Regev, Scalable querying of human cell atlases via a foundational model reveals commonalities across fibrosis-associated macrophages. *bioRxiv* , 2023.07.18.549537 (2023).](https://www.zotero.org/google-docs/?9eqRuI)

[6. L. Kong, V. Pokatayev, A. Lefkovith, G. T. Carter, E. A. Creasey, C. Krishna, S. Subramanian, B. Kochar, O. Ashenberg, H. Lau, A. N. Ananthakrishnan, D. B. Graham, J. Deguine, R. J. Xavier, The landscape of immune dysregulation in Crohn’s disease revealed through single-cell transcriptomic profiling in the ileum and colon. *Immunity* **56**, 444-458.e5 (2023).](https://www.zotero.org/google-docs/?9eqRuI)

[7. J. C. Martin, C. Chang, G. Boschetti, R. Ungaro, M. Giri, J. A. Grout, K. Gettler, L. Chuang, S. Nayar, A. J. Greenstein, M. Dubinsky, L. Walker, A. Leader, J. S. Fine, C. E. Whitehurst, M. L. Mbow, S. Kugathasan, L. A. Denson, J. S. Hyams, J. R. Friedman, P. T. Desai, H. M. Ko, I. Laface, G. Akturk, E. E. Schadt, H. Salmon, S. Gnjatic, A. H. Rahman, M. Merad, J. H. Cho, E. Kenigsberg, Single-Cell Analysis of Crohn’s Disease Lesions Identifies a Pathogenic Cellular Module Associated with Resistance to Anti-TNF Therapy. *Cell* **178**, 1493-1508.e20 (2018).](https://www.zotero.org/google-docs/?9eqRuI)

[8. C. S. Smillie, M. Biton, J. Ordovas-Montanes, K. M. Sullivan, G. Burgin, D. B. Graham, R. H. Herbst, N. Rogel, M. Slyper, J. Waldman, M. Sud, E. Andrews, G. Velonias, A. L. Haber, K. Jagadeesh, S. Vickovic, J. Yao, C. Stevens, D. Dionne, L. T. Nguyen, A.-C. Villani, M. Hofree, E. A. Creasey, H. Huang, O. Rozenblatt-Rosen, J. J. Garber, H. Khalili, A. N. Desch, M. J. Daly, A. N. Ananthakrishnan, A. K. Shalek, R. J. Xavier, A. Regev, Intra- and Inter-cellular Rewiring of the Human Colon during Ulcerative Colitis. *Cell* **178**, 714-730.e22 (2019).](https://www.zotero.org/google-docs/?9eqRuI)

[9. M. D. Robinson, D. J. McCarthy, G. K. Smyth, edgeR: a Bioconductor package for differential expression analysis of digital gene expression data. *Bioinformatics* **26**, 139–140 (2010).](https://www.zotero.org/google-docs/?9eqRuI)

[10. I. Korsunsky, A. Nathan, N. Millard, S. Raychaudhuri, Presto scales Wilcoxon and auROC analyses to millions of observations. *bioRxiv* , 653253 (2019).](https://www.zotero.org/google-docs/?9eqRuI)

[11. J. Cao, M. Spielmann, X. Qiu, X. Huang, D. M. Ibrahim, A. J. Hill, F. Zhang, S. Mundlos, L. Christiansen, F. J. Steemers, C. Trapnell, J. Shendure, The single-cell transcriptional landscape of mammalian organogenesis. *Nature* **566**, 496–502 (2019).](https://www.zotero.org/google-docs/?9eqRuI)

[12. X. Qiu, Q. Mao, Y. Tang, L. Wang, R. Chawla, H. A. Pliner, C. Trapnell, Reversed graph embedding resolves complex single-cell trajectories. *Nat. Methods* **14**, 979–982 (2017).](https://www.zotero.org/google-docs/?9eqRuI)

[13. C. Trapnell, D. Cacchiarelli, J. Grimsby, P. Pokharel, S. Li, M. Morse, N. J. Lennon, K. J. Livak, T. S. Mikkelsen, J. L. Rinn, The dynamics and regulators of cell fate decisions are revealed by pseudotemporal ordering of single cells. *Nat. Biotechnol.* **32**, 381–386 (2014).](https://www.zotero.org/google-docs/?9eqRuI)

[14. S. Jin, C. F. Guerrero-Juarez, L. Zhang, I. Chang, R. Ramos, C.-H. Kuan, P. Myung, M. V. Plikus, Q. Nie, Inference and analysis of cell-cell communication using CellChat. *Nat. Commun.* **12**, 1088 (2021).](https://www.zotero.org/google-docs/?9eqRuI)

[15. R. Casadio, E. Frigimelica, P. Bossù, D. Neumann, M. U. Martin, A. Tagliabue, D. Boraschi, Model of interaction of the IL‐1 receptor accessory protein IL‐1RAcP with the IL‐1β/IL‐1RI complex. *FEBS Lett.* **499**, 65–68 (2001).](https://www.zotero.org/google-docs/?9eqRuI)
